# Frequency of use and characterization of frailty assessments in observational studies on older women with breast cancer: a systematic review

**DOI:** 10.1186/s12877-024-05152-5

**Published:** 2024-06-27

**Authors:** Dafne N. Sanchez, Marloes G. M. Derks, Jose A. Verstijnen, Dominik Menges, Johanneke E. A. Portielje, Frederiek Van den Bos, Esther Bastiaannet

**Affiliations:** 1 Epidemiology, Biostatistics and Prevention Institute (EBPI), University of Zürich, Hirschengraben 82, Zurich, CH-8001 Switzerland; 2 Department of Medical Oncology, Leiden University Medical Center, Leiden, The Netherlands; 3 Department of Medical Oncology, Maasstad Hospital, Rotterdam, The Netherlands; 4 Department of Gerontology and Geriatrics, Leiden University Medical Center, Leiden, The Netherlands

**Keywords:** Frailty, Breast neoplasms, Systematic review, Elderly health, Geriatric assessment

## Abstract

**Background:**

Breast cancer and frailty frequently co-occur in older women, and frailty status has been shown to predict negative health outcomes. However, the extent to which frailty assessments are utilized in observational research for the older breast cancer population is uncertain. Therefore, the aim of this review was to determine the frequency of use of frailty assessments in studies investigating survival or mortality, and characterize them, concentrating on literature from the past 5 years (2017–2022).

**Methods:**

MEDLINE, EMBASE and Cochrane Library were systematically queried to identify observational studies (case-control, cohort, cross-sectional) published from 2017-2022 that focus on older females (*≥ *65 years) diagnosed with breast cancer, and which evaluate survival or mortality outcomes. Independent reviewers assessed the studies for eligibility using Covidence software. Extracted data included characteristics of each study as well as information on study design, study population, frailty assessments, and related health status assessments. Risk of bias was evaluated using the appropriate JBI tool. Information was cleaned, classified, and tabulated into review level summaries.

**Results:**

In total, 9823 studies were screened for inclusion. One-hundred and thirty studies were included in the final synthesis. Only 11 (8.5%) of these studies made use of a frailty assessment, of which 4 (3.1%) quantified frailty levels in their study population, at baseline. Characterization of frailty assessments demonstrated that there is a large variation in terms of frailty definitions and resulting patient classification (i.e., fit, pre-frail, frail). In the four studies that quantified frailty, the percentage of individuals classified as pre-frail and frail ranged from 18% to 29% and 0.7% to 21%, respectively. Identified frailty assessments included the Balducci score, the Geriatric 8 tool, the Adapted Searle Deficits Accumulation Frailty index, the Faurot Frailty index, and the Mian Deficits of Accumulation Frailty Index, among others. The Charlson Comorbidity Index was the most used alternative health status assessment, employed in 56.9% of all 130 studies. Surprisingly, 31.5% of all studies did not make use of any health status assessments.

**Conclusion:**

Few observational studies examining mortality or survival outcomes in older women with breast cancer incorporate frailty assessments. Additionally, there is significant variation in definitions of frailty and classification of patients. While comorbidity assessments were more frequently included, the pivotal role of frailty for patient-centered decision-making in clinical practice, especially regarding treatment effectiveness and tolerance, necessitates more deliberate attention. Addressing this oversight more explicitly could enhance our ability to interpret observational research in older cancer patients.

**Supplementary Information:**

The online version contains supplementary material available at 10.1186/s12877-024-05152-5.

## Introduction

Female breast cancer (BC) has surpassed lung cancer as the most commonly diagnosed cancer across the globe. At the same time, trends in the burden of breast cancer, measured by incidence and mortality, have continued to increase steadily [1]. GLOBOCAN estimates produced by the International Agency for Research on Cancer (IARC) revealed 2.3 million new cases of breast cancer worldwide, which accounted for 11.7% of all new cancer cases, and 685,000 deaths in 2020 [2]. Given that aging is the largest risk factor for breast cancer, older women develop BC at higher incidence rates compared to their younger counterparts [3]. Furthermore, as population life expectancy improves, the number of older women living with breast cancer is expected to rise.

Evidence supports the need for differential, tailored treatment between younger and older BC patients [4–9]. Clinical decision-making for the older cancer patient population (65+) is especially challenging because it is heterogeneous in nature and must take into account additional relevant factors such as frailty, multimorbidity, polypharmacy, limited life expectancy, and correspondingly death from competing causes besides the cancer of interest [10, 11]. However, these factors often lead to clinical study exclusions [12]. As a result, older women have been largely underrepresented in randomized clinical trials, therefore leading to a lack of evidence-based information on the best treatment within these age groups and a heavy reliance on observational research [13]. The prevalence of frailty increases with advancing age and more than 50% of older cancer patients are considered pre-frail or frail [14].

The notion of frailty has been historically difficult to capture considering its manifestation is highly complex and any underlying pathophysiological mechanisms are multifactorial [15]. Frailty is theoretically defined as an age-related syndrome of physiological decline and vulnerability, leading to an increased risk of adverse health outcomes [16–19]. Frailty has also been defined and quantified using several methods, two of which are particularly well-known and used in both clinical and research settings: the Frailty Phenotype [20] and the Frailty Index (FI ) [21]. The frailty phenotype by Fried and colleagues defines frailty as a condition meeting 3 of 5 phenotypic criteria, while the frailty index defines frailty through the proportion of accumulated deficits.

Many healthcare practitioners advocate for older adults to be evaluated via Comprehensive geriatric assessment (CGA), which is a multidimensional, multidisciplinary process which identifies their medical, social and functional needs, and supports the development of a care plan to address those needs [22]. In the field of geriatric oncology, CGA is used to detect disabilities, and conditions that potentially contribute to an older patient’s frailty status, which could predispose them to poor outcomes and treatment complications [23–25]. Furthermore, the insights gained from CGA can inform the coordination and planning of interventions designed to mitigate the impact of frailty on cancer treatment outcomes. CGA is often criticized for being time consuming, requiring the need for coordination of multidisciplinary specialties, and lacking consistency in collected data [26]. As a result, many cancer specialists seek a shorter screening tool that can separate fit older cancer patients, eligible for standard cancer treatment, from vulnerable patients who should subsequently receive a full assessment to guide tailoring of their treatment regimens. Additionally, although CGA can provide a comprehensive overview of a patient’s vulnerabilities, it alone does not provide a numerical measurement of frailty and must be operationalized on a scale or index for use in outcomes research [27].

Closely related concepts to frailty such as comorbidity and disability, as well as various geriatric parameters have been similarly utilized to characterize the health status of older breast cancer patients and have been shown to predict disease related survival, toxicity, patient reported outcomes (PROs), and mortality [28]. While comorbidity, disability, and other geriatric parameters can contribute to the development of frailty, it is crucial to recognize that frailty itself is a distinct and vital entity that holds paramount importance in the treatment of older cancer patients. Notably, frailty represents an aggregate expression of risk [29] that extends beyond the presence of individual conditions, and is considered preventable and partially reversable [30, 31].

Given the value of frailty assessments, it is crucial to understand their use in breast cancer research. To date, no reviews have yet quantified the use of frailty assessments in observational studies on breast cancer in older women. Therefore, the aim of this review was to determine the frequency of use of frailty assessments in such studies and characterize them, concentrating on literature from the past 5 years (2017-2022). The 5-year timeline was considered suitable since the intention was to capture current research practices.

## Methods

This systematic review followed the PRISMA guidelines [32] (Preferred Reporting Items for Systematic reviews and Meta-Analyses). A protocol was developed a priori; however, it was not registered or published (see Appendix A.1). The specific objectives of this review were as follows:

Primary objectives:

1. Quantify and characterize frailty assessments in included observational studies.
2. Document which observational studies have been published in the last 5 years (2017–2022)

Secondary objectives:

1. Assess the prevalence of frailty in older breast cancer patients

###  Search strategy and article selection

A systematic literature search was conducted to identify observational studies on older women with breast cancer reporting survival or mortality. Literature published from 2017-2022 was retrieved from 3 databases including: MEDLINE, EMBASE, and Cochrane Library. Additional articles were mined by searching on Google Scholar and inspecting reference lists of relevant systematic reviews. The search strategy can be accessed in Appendix A.1.

Studies were eligible for inclusion if they fulfilled the following criteria:

1. Article was (or reported on) an observational study defined here as a case–control study, cross-sectional study, or cohort study.
2. Article reported solely on older females ≥ 65 years of age with all stages of breast cancer who were patients receiving active oncological treatment at the time of enrollment.
3. Article was written in English, German, Dutch, or Spanish.
4. Article reported on survival or mortality before or after treatment.
5. Article was published within the specified 5-year period (2017–2022)

Studies were excluded based the following criteria:

1. Article was a letter, comment, conference abstract, partial text, or review.
2. Article reported on a mixed population which includes individuals younger than 65 years of age, male patients, cancers besides breast cancer, and patients receiving best supportive care without active oncological treatment in the last stage of the disease.
3. Article was about health technology assessment, (population) breast cancer screening, or a tool validation study.
4. Article was primarily a molecular analysis (i.e., RNA, DNA, tumor structure, single cells, protein expression, biomarkers, genomic testing, gene expression etc.)

The list of excluded articles can be accessed in Appendix A.2.

###  Data retrieval, extraction, and synthesis

Collected references were managed using Covidence Software [33]. Duplicate articles were removed prior to the start of the review process. Eligibility of identified studies was determined by independently assessing titles and abstracts by two authors including DS, EB, MD, DM, FB, JP, or JV. Subsequently, the full texts of selected articles were independently assessed by DS and EB. Any disagreements on inclusion were resolved by consensus by DS and EB. A data extraction form was developed using Covidence, pilot tested on 10 randomly selected articles, and refined prior to use. Two unique sets of extracted data were independently collected (DS and EB) for each article and consolidated into a final version to ensure agreement and completeness. Extracted data included characteristics of each study such as title, DOI, country of publication, inclusion/exclusion criteria, aim, outcomes, study design, and funding sources. We also collected information on the population such as the number of patients used in the analysis, number of fit/pre-frail/frail patients, age, cancer stages and treatments, information on the use of frailty assessments, comorbidity assessments, or related health status assessments, as well as data source and setting. Variables were cleaned, classified, and tabulated into review level summaries for interpretation. Cancer stage, often described by TNM, or other stage descriptors were categorized to non-invasive non-metastatic, invasive non-metastatic, invasive metastatic, or unclear for simplicity. Descriptive statistics were performed using R (version 4.2.1, R Core Team, 2022) and Rstudio (version 2023.3.0.386, RStudio Team, 2023), while tables and figures were generated with the following attached packages: ggplot2 3.3.6, xtable 1.8-4, dplyr 1.0.9, and readr 2.1.2.

###  Quality assessment

Risk of bias was assessed separately by EB and DS using critical appraisal tools from the Joanna Briggs Institute (JBI) [34]. The appropriate checklist was selected per observational study type. Each checklist is composed of several questions answered as “yes”, “unclear”, “no”, or “not applicable”. Any disagreements were solved by consensus. Studies were labeled low, medium, or high risk of bias based on the applicable questions.

## Results

### Literature search and inclusion

**Fig. 1:**
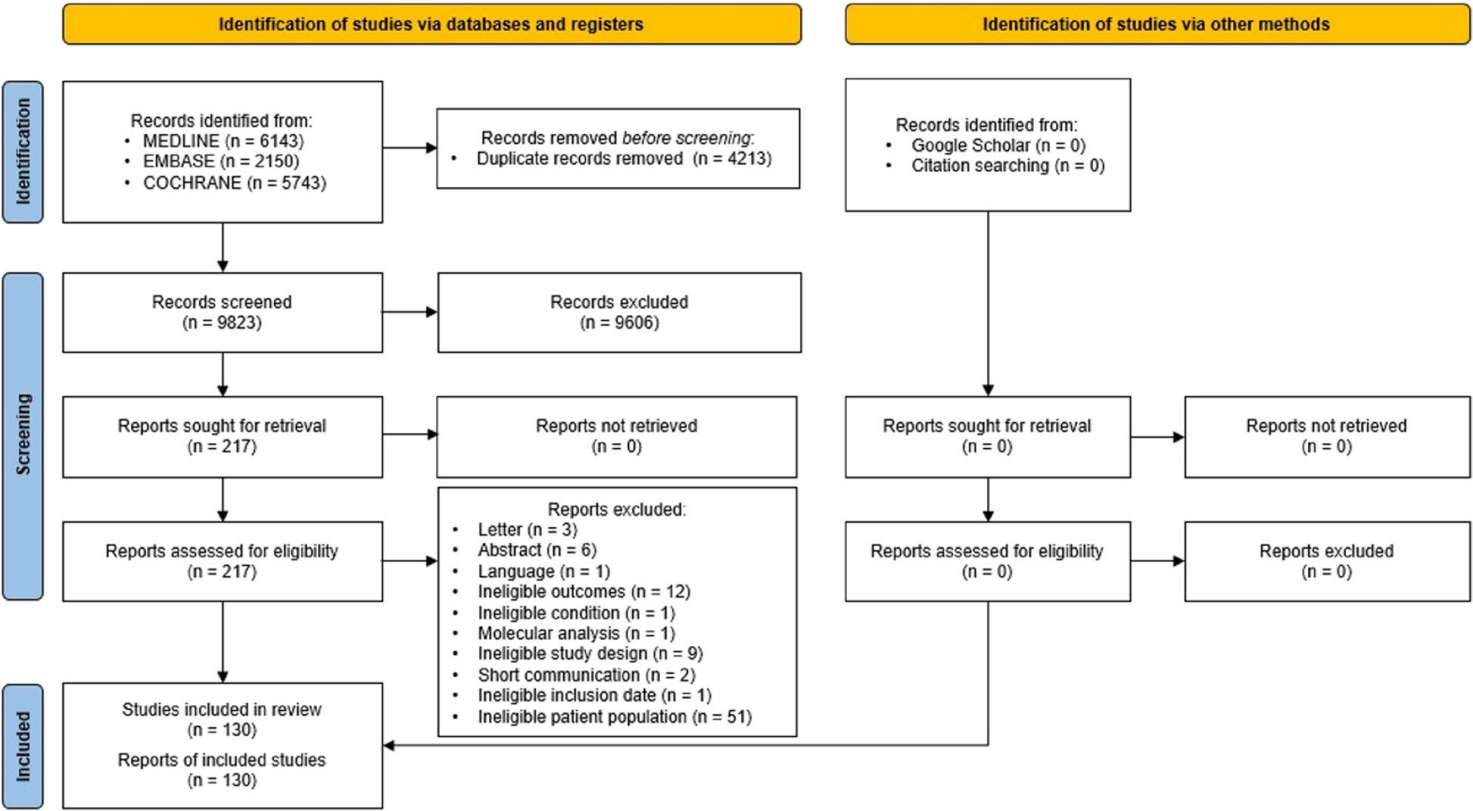
Results of the search strategy and reasons for exclusion

The search strategy yielded 14,036 records. After removing duplicate records, 9283 were screened on their titles and abstracts. Following this screening, 217 studies were deemed potentially eligible and were reviewed in full-text. Out of these, 130 studies met the inclusion criteria and were included in the systematic review. The PRISMA Flow Diagram (Fig. 1) shows an overview of the study selection and reasons for exclusion.

## Study characteristics

From the 130 included studies, 71 used data from North America, 39 from Europe, 13 from Asia, 5 from Europe and Asia, 1 from North America and Asia, and 1 from Europe and North America. One-hundred twenty-eight were cohort studies (114 retrospective studies, 14 prospective studies), 1 was a retrospective case-control study, and 1 was a cross-sectional study. Fifty-six studies had a minimum age under 70 years and 73 had a minimum age above 70 years. Ninety-nine studies examined patients with invasive non- metastatic cancer, 8 with invasive metastatic cancer, 1 with non-invasive non-metastatic cancer, 15 examined a combination of invasive metastatic, invasive non-metastatic, and non-invasive non-metastatic cancers, and the remaining 7 were unclear. Patient data stemmed from various sources; however, the majority were from single cancer registries or institutional databases. The complete overview of study characteristics is detailed in Table 1.

Due to the nature of the review, all studies were included in the synthesis. Risk of bias was assessed for 130 studies using the appropriate JBI Critical Appraisal tool. The quality of the studies was mixed; however, all were determined to have low or medium risk of bias overall. Full details of the risk of bias assessment are displayed in Appendix A.3.

**Table 1:** Characteristics of included studies

Study ID	Country	Study design	Data source	Stage	Frailty assessed	No. in analysis	Age	Setting
Agborbesong 2020 [35]	US	R CS; Single center	Institutional records or database	Non-invasive non-metastatic; Invasive non-metastatic	No	179	70 +	North America
Akushevich 2020 [36]	US	R CS; NA	Cancer registry linked to an administrative database	Non-invasive non-metastatic	No	22,576	65 +	North America
Alatawi 2021 [37]	SA	R CS; NA	Cancer registry linked to an administrative database	Invasive non-metastatic; Invasive metastatic	No	11,084	67 +	North America
Ali 2019 [38]	US	R CS; NA	Cancer registry linked to an administrative database	Invasive non-metastatic	No	5688	65 +	North America
Al-Rashdan 2021 [39]	CA	R CS; NA	Administrative database; Cancer registry; Census	Invasive non-metastatic	No	1369	80 +	North America
Aly 2019 [40]	US	R CS; NA	Cancer registry linked to an administrative database	Invasive metastatic	No	625	66 +	North America
Aytekin 2017 [41]	TR	R CS; Single center	Institutional records or database	Invasive non-metastatic	No	238	70 +	Europe; Asia
Battisti 2021 [42]	UK	P CS; Multicenter	Cohort study	Invasive non-metastatic	Yes	2756	70 +	Europe
Bertolo 2020 [43]	CA	R CS; Single center	Institutional records or database	Invasive non-metastatic; Invasive metastatic	No	97	80 +	North America
Blanchette 2020 [44]	CA	R CS; NA	Administrative database	Invasive non-metastatic	No	5692	66 +	North America
Blay Aulina 2022 [45]	ES	R CS; Single center	Institutional records or database	Non-invasive non-metastatic; Invasive non-metastatic; Invasive metastatic	No	63	80 +	Europe
Buszek 2019 [46]	US	R CS; Multicenter	Cancer registry	Invasive non-metastatic	No	2995	70 +	North America
Cao 2018 [47]	FR	R CS; Single center	Institutional records or database	Invasive non-metastatic	No	752	70 +	Europe
Chadha 2019 [48]	US	R CS; Single center	Institutional records or database	Invasive non-metastatic	No	92	65 +	North America
Chagpar 2017 [49]	US	R CS; NA	Cancer registry	Unclear	No	157,584	70 +	North America
Chen 2018 [50]	US	R CS; NA	Cancer registry	Invasive non-metastatic	No	635	75 +	North America
Chen 2021 [51]	CN	R CS; NA	Cancer registry	Invasive metastatic	No	1801	70 +	North America
Chu 2018 [52]	US	R CS; NA	Cancer registry	Invasive non-metastatic	No	16,362	70 +	North America
Cil 2022 [53]	TR	R CS; Multicenter	Institutional records or database	Unclear	No	93	65 +	Europe; Asia
Corso 2021 [54]	IT	R CS; NA	Institutional records or database	Invasive non-metastatic	No	252	70 +	Europe
Crozier 2020 [55]	US	R CS; NA	Cancer registry	Invasive non-metastatic	No	1884	70 +	North America
Dahn 2020 [56]	CA	R CS; NA	Cancer registry	Invasive non-metastatic	No	460	70 +	North America
De Boer 2020 [57]	NL	R CS; Single center	Institutional records or database	Invasive non-metastatic	No	2200	70 +	Europe; North America
De Boer 2021 [28]	NL	R CS; NA	Cancer registry	Invasive non-metastatic	No	7511	70 +	Europe
De Luca 2021 [58]	IT	R CS; Single center	Institutional records or database	Invasive metastatic	No	40	70 +	Europe
Derks 2018 [59]	NL	R CS; NA	Cancer registry	Invasive non-metastatic	No	236,015	70 +	Europe
De Santis 2018 [60]	IT	P CS; Single center	Institutional records or database	Invasive non-metastatic	No	752	65 +	Europe
Downs-Canner 2019 [61]	US	P CS; Single center	Institutional records or database	Invasive non-metastatic	No	323	70 +	North America
Du 2022 [62]	US	R CS; NA	Cancer registry linked to an administrative database	Non-invasive non-metastatic; Invasive non-metastatic; Invasive metastatic	No	92,829	65 +	North America
Dumontier 2017 [63]	US	P CS; Multicenter	Cancer registry; Institutional records or database	Invasive non-metastatic	No	660	65 +	North America
El Badri 2021 [64]	UK	R CS; Multicenter	Institutional records or database	Unclear	No	276	75 +	Europe
Enomoto 2021 [65]	JP	R CS; Single center	Institutional records or database	Invasive non-metastatic	No	60	85 +	North America; Asia
Escott 2020 [66]	US	R CS; Multicenter	Cancer registry	Invasive non-metastatic	No	12,036	70 +	North America
Faiz 2018 [67]	US	R CS; NA	Cancer registry linked to a health outcomes database	Non-invasive non-metastatic; Invasive non-metastatic	No	276,028	65 +	North America
Fattoruso 2022 [68]	IT	R CS; Single center	Institutional records or database	Invasive metastatic	No	84	70 +	Europe
Frebault 2022 [69]	US	R CS; Multicenter	Cancer registry	Invasive non-metastatic	No	62,575	80 +	North America
Gal 2018 [70]	IL	R CS; Single center	Institutional records or database	Invasive non-metastatic	No	390	65 +	Asia
Goldberg 2019 [71]	CA	R CS; Multicenter	Research database	Invasive non-metastatic	No	5076	65 +	North America
Goyal 2019 [72]	US	R CS; NA	Cancer registry linked to a health outcomes database	Invasive metastatic	No	3622	66 +	North America
Hannoun-Levi 2021 [73]	FR	R CS; Single center	Institutional records or database	Invasive non-metastatic	Yes	157	70 +	Europe
Haque 2017 [74]	US	R CS; NA	Cancer registry	Invasive non-metastatic	No	121,312	70 +	North America
Haque 2018 [75]	US	R CS; NA	Cancer registry	Invasive non-metastatic	No	547	70 +	North America
Haque 2019 [76]	US	R CS; NA	Cancer registry	Invasive non-metastatic	No	8631	70 +	North America
Herskovic 2018 [77]	US	R CS; NA	Cancer registry	Invasive non-metastatic	No	61,395	65 +	North America
Hornova 2017 [78]	CZ	R CS; Single center	Institutional records or database	Invasive non-metastatic	No	80	70 +	Europe
Huang 2022 [79]	CN	R CS; NA	Cancer registry	Invasive non-metastatic	No	4696	70 +	North America
Iglay 2017 [80]	US	R CS; NA	Cancer registry linked to a health outcomes database	Invasive non-metastatic	No	19,028	68 +	North America
Janeva 2020 [81]	SE	R CS; NA	Research database	Invasive non-metastatic	No	1130	70 +	Europe
Jhawar 2020 [82]	US	R CS; NA	Cancer registry	Invasive non-metastatic	No	130,194	65 +	North America
Jobsen 2019 [83]	NL	R CS; Single center	Cancer registry	Invasive non-metastatic	No	1425	65 +	Europe
Jobsen 2021 [84]	NL	R CS; Single center	Cancer registry	Invasive non-metastatic	No	1205	65 +	Europe
Karanlik 2017 [85]	TR	R CCS; Single center	Institutional records or database	Invasive non-metastatic	No	91	65 +	Europe; Asia
Kedzierawski 2021 [86]	PL	R CS; Single center	Institutional records or database	Invasive non-metastatic	Yes	259	75 +	Europe
Kinj 2018 [87]	FR	R CS; Single center	Institutional records or database	Invasive non-metastatic	No	48	65 +	Europe
Kinj 2019 [88]	FR	R CS; Single center	Institutional records or database	Invasive non-metastatic	No	48	65 +	Europe
Klint 2021 [89]	SE	R CS; Single center	Institutional records or database	Invasive non-metastatic; Invasive metastatic	No	115	70 +	Europe
Kong 2018 [90]	US	R CS; NA	Cancer registry linked to an administrative database	Invasive non-metastatic	No	27,706	66 +	North America
La Rocca 2020 [91]	IT	P CS; Single center	Institutional records or database	Invasive non-metastatic	No	794	65 +	Europe
La Rocca 2020 [92]	IT	P CS; Single center	Institutional records or database	Invasive non-metastatic	No	735	65 +	Europe
Leo 2019 [93]	IT	P CS; Multicenter	Institutional records or database	Invasive metastatic	Yes	50	65 +	Europe
Lin 2021 [94]	TW	R CS; Single center	Institutional records or database	Invasive non-metastatic; Invasive metastatic	No	503	65 +	Asia
Liu 2021 [95]	CN	R CS; Single center	Institutional records or database	Invasive non-metastatic	No	1094	65 +	Asia
Luo 2020 [96]	CN	R CS; NA	Cancer registry	Invasive non-metastatic	No	75,950	70 +	North America
Luo 2021 [97]	CN	R CS; NA	Cancer registry	Invasive non-metastatic	No	6494	70 +	North America
Mandelblatt 2017 [98]	US	P CS; Multicenter	Clinical trials database	Invasive non-metastatic	Yes	1265	65 +	North America
Marks 2020 [99]	US	R CS; NA	Cancer registry	Invasive non-metastatic	No	9026	70 +	North America
Martin 2021 [100]	UK	P CS; Multicenter	Cohort study	Invasive non-metastatic	No	3416	70 +	Europe
McKevitt 2021 [101]	CA	R CS; Multicenter	Institutional records or database	Unclear	No	2662	70 +	North America
Mermut 2019 [102]	TR	R CS; Single center	Institutional records or database	Invasive non-metastatic; Invasive metastatic	No	148	70 +	Europe; Asia
Merrill 2017 [103]	US	R CS; Single center	Institutional records or database	Invasive non-metastatic	No	92	80 +	North America
Mogal 2017 [104]	US	R CS; NA	Cancer registry linked to a health outcomes database	Invasive non-metastatic	No	1784	70 +	North America
Morgan 2020 [105]	UK	P CS; Multicenter	Cohort study	Invasive non-metastatic	Yes	2816	70 +	Europe
Morita 2022 [106]	JP	R CS; Multicenter	Institutional records or database	Invasive non-metastatic	No	905	70 +	Asia
Nayyar 2020 [107]	US	R CS; NA	Cancer registry linked to an administrative database	Invasive non-metastatic	No	8784	70 +	North America
Nichol 2017 [108]	CA	R CS; NA	Research database	Invasive non-metastatic	No	722	70 +	North America
Ogawa 2019 [109]	JP	R CS; Single center	Institutional records or database	Invasive non-metastatic	No	170	75 +	Asia
Ojala 2019 [110]	FI	R CS; Single center	Institutional records or database	Invasive non-metastatic	No	446	80 +	Europe
Oktay 2019 [111]	TR	R CS; Multicenter	Institutional records or database	Invasive non-metastatic	No	87	65 +	Europe; Asia
Onega 2018 [112]	US	R CS; Multicenter	Research database	Invasive non-metastatic	No	4454	66 +	North America
Park 2017 [113]	KR	R CS; Multicenter	Institutional records or database	Invasive metastatic	No	161	65 +	Asia
Park 2022 [114]	US	CSS; NA	Cancer registry linked to a health outcomes database	Invasive non-metastatic	No	3537	65 +	North America
Peng 2021 [115]	CN	R CS; Single center	Institutional records or database	Invasive non-metastatic; Invasive metastatic	No	420	70 +	Asia
Pinsky 2020 [116]	US	R CS; NA	Cancer registry linked to an administrative database	Invasive non-metastatic; Invasive metastatic	No	117,840	65 +	North America
Poodt 2018 [117]	NL	R CS; Multicenter	Cancer registry	Invasive non-metastatic	No	1467	75 +	Europe
Rais 2021 [118]	CA	R CS; Single center	Institutional records or database	Invasive non-metastatic	No	50	NA	North America
Reeder-Hayes 2017 [119]	US	R CS; NA	Cancer registry linked to an administrative database	Invasive non-metastatic	No	416	66 +	North America
Reeder-Hayes 2021 [120]	US	R CS; NA	Cancer registry and Research database	Invasive non-metastatic	Yes	10,204	66 +	North America
Ring 2021 [121]	UK	P CS; Multicenter	Cohort study	Invasive non-metastatic	Yes	2811	70 +	Europe
Schuil 2018 [122]	NL	R CS; Multicenter	Cancer registry	Invasive non-metastatic	No	3619	70 +	Europe
Schwartz 2018 [123]	US	R CS; NA	Cancer registry linked to an administrative database	Invasive non-metastatic; Invasive metastatic	No	1244	66 +	North America
Showalter 2021 [124]	US	R CS; NA	Cancer registry linked to an administrative database	Invasive non-metastatic	No	10,719	70 +	North America
Sieluk 2021 [125]	US	R CS; NA	Cancer registry linked to a health outcomes database	Invasive non-metastatic	No	1569	65 +	North America
Smith-Graziani 2020 [126]	US	R CS; NA	Cancer registry	Invasive non-metastatic	No	28,968	66 +	North America
Stueber 2020 [127]	DE	R CS; Multicenter	Institutional records or database	Invasive non-metastatic	No	2384	70 +	Europe
Suarez-Almazor 2020 [128]	US	R CS; NA	Administrative database; Cancer registry	Invasive non-metastatic	No	37,724	66 +	North America
Suen 2020 [129]	CN	R CS; Single center	Institutional records or database	Invasive non-metastatic	No	357	70 +	Asia
Sumodhee 2017 [130]	FR	R CS; Single center	Institutional records or database	Invasive non-metastatic	No	79	66 +	Europe
Sun 2021 [131]	US	R CS; Single center	Clinical database	Invasive non-metastatic	No	500	70 +	North America
Takada 2019 [132]	JP	R CS; Single center	Institutional records or database	Unclear	No	75	65 +	Asia
Tamirisa 2018 [133]	US	R CS; NA	Cancer registry	Invasive non-metastatic	No	133,778	70 +	North America
Tamirisa 2020 [134]	US	R CS; NA	Cancer registry	Invasive non-metastatic	No	592	70 +	North America
Tamirisa 2021 [135]	US	R CS; NA	Cancer registry	Invasive non-metastatic	No	1972	70 +	North America
Tang 2018 [136]	US	R CS; Multicenter	Administrative database	Unclear	No	5969	67 +	North America
Tang 2021 [137]	JP	R CS; Single center	Institutional records or database	Invasive non-metastatic	No	170	65 +	Asia
Tang 2022 [138]	CN	R CS; NA	Cancer registry	Invasive non-metastatic	No	4761	70 +	North America
Tannenbaum 2017 [139]	US	R CS; NA	Cancer registry linked to an administrative database	Invasive non-metastatic	No	12,610	67 +	North America
Thompson 2021 [140]	US	R CS; Single center	Cancer registry; Institutional records or database	Invasive non-metastatic	No	487	70 +	North America
Tringale 2021 [141]	US	R CS; Single center	Institutional records or database	Invasive non-metastatic	No	888	65 +	North America
Valachis 2021 [142]	SE	R CS; NA	Research database	Invasive non-metastatic	No	413	70 +	Europe
Valli 2018 [143]	CH	R CS; Single center	Institutional records or database	Invasive non-metastatic	No	137	70 +	Europe
Van der Plas-Krijgsman 2022 [144]	NL and UK	P CS; Multicenter	Cohort study	Invasive non-metastatic	Yes	3880	70 +	Europe
Vyas 2021 [145]	US	R CS; NA	Cancer registry linked to an administrative database	Invasive metastatic	No	1282	66 +	North America
Wang 2018 [146]	CN	R CS; NA	Cancer registry	Invasive non-metastatic; Invasive metastatic	No	5068	80 +	North America
Ward 2018 [147]	UK	R CS; NA	Cancer registry	Invasive non-metastatic; Invasive metastatic	No	23,849	70 +	Europe
Ward 2019 [148]	UK	R CS; Multicenter	Cancer registry	Invasive non-metastatic	No	11,735	70 +	Europe
Wasif 2019 [149]	US	R CS; NA	Cancer registry linked to an administrative database	Invasive non-metastatic	No	47,220	65 +	North America
Wickberg 2018 [150]	SE	P CS; Multicenter	Institutional records or database	Invasive non-metastatic	No	603	65 +	Europe
Wittayanukorn 2018 [151]	US	R CS; NA	Cancer registry linked to a health outcomes database	Invasive non-metastatic; Invasive metastatic	No	6542	66 +	North America
Wu 2019 [152]	CN	R CS; NA	Cancer registry; Research database	Invasive non-metastatic	No	3072	65 +	North America
Wu 2019 [153]	CN	R CS; NA	Cancer registry	Invasive non-metastatic	No	2020	65 +	North America
Wyld 2021 [154]	UK	P CS; Multicenter	Cohort study	Invasive non-metastatic	Yes	660	70 +	Europe
Yan 2021 [155]	US	R CS; NA	Cancer registry linked to a health outcomes database	Invasive non-metastatic	Yes	2411	65 +	North America
Yang 2021 [156]	CN	R CS; NA	Cancer registry	Invasive non-metastatic	No	28,068	65 +	North America
Yuan 2020 [157]	US	R CS; NA	Cancer registry linked to a health outcomes database	Invasive non-metastatic	No	552	70 +	North America
Zanuso 2020 [158]	IT	R CS; Single center	Institutional records or database	Unclear	No	128	65 +	Europe
Zhao 2021 [159]	ES	R CS; Single center	Institutional records or database	Invasive non-metastatic	No	47	70 +	Europe
Zhi 2019 [160]	CN	R CS; Single center	Institutional records or database	Invasive non-metastatic	No	327	65 +	Asia
Zhong 2020 [161]	CN	R CS; Single center	Institutional records or database	Invasive non-metastatic	No	481	70 +	Asia
Zhong 2020 [162]	CN	R CS; Single center	Institutional records or database	Invasive non-metastatic	No	450	70 +	Asia
Zhou 2018 [163]	US	R CS; NA	Cancer registry	Invasive non-metastatic	No	53,950	70 +	North America

*NA *Not available, *R CS* Retrospective Cohort Study,* P CS* Prospective Cohort Study, *CSS* Cross Sectional Study, R *CCS* Retrospective Case Control Study

## Frailty assessments

Eleven studies [42, 73, 86, 93, 98, 105, 120, 121, 144, 154, 155] (8.5% of 130 included studies) assessed frailty in their patient population, however only 4 studies [42, 98, 121, 155] classified patients into fit, pre-frail, or frail categories. Frailty was only assessed at baseline and there were no studies which assessed frailty post-treatment. Patients in each study included those treated with surgery, radiotherapy, hormonal therapy, chemotherapy, or targeted therapy. The assessments included the Balducci Score, the Geriatric 8 tool, the CGA, the Adapted Searle Deficits of Accumulations Index, Activities of Daily Living/Instrumental Activities of Daily Living, the Faurot Frailty Index, the Mian Deficits of Accumulations Index, and various combinations of geriatric tests. The identified frailty assessments were highly heterogeneous in terms of their operationalization, definitions, and patient classification. In total, there were ten unique definitions of frailty from eleven studies. Surprisingly, 4 studies [42, 121, 144, 154] identified frailty using a novel definition based off select geriatric assessments. One study [105] used Activities of Daily Living and Instrumental Activities of Daily Living to define and assess frailty.

The most common approaches to operationalizing frailty included the use of scores, binary scales, or indices. However, it was observed that the results of these frailty measurements were frequently either not reported or not utilized in subsequent analyses or interpretations within the studies. Furthermore, all identified frailty assessments incorporated at least one of two key components in their definition of frailty: comorbidity and functional status, with the latter most always encompassing disability. In addition to these core elements, many frailty assessments also included other geriatric parameters, such as cognitive function, nutritional status, polypharmacy, as well as various others.

Among the four studies which quantified frailty, the percentage of pre-frail individuals ranged from 18 to 29 percent, while the percentage of frail individuals ranged from 0.7 to 21 percent (percentage of frail patients was not reported by 7 studies). Two of these studies [42, 121] operationally defined frailty using a novel index based on seven geriatric assessments (Charlson Comorbidity Index, Activities of Daily Living, Instrumental Activities of Daily Living, Eastern Cooperative Oncology Group (ECOG) Performance Status, Mini Mental State Examination, and Abridged Patient-Generated Subjective Global Assessment), and the remaining two used established indices, namely the Adapted Searle Deficits Accumulation Frailty Index [98], and the Mian Deficits of Accumulation Frailty Index [155]. A summary of characteristics including details on the domains and geriatric parameters which define each assessment is indicated in Table 2. Author provided frailty definitions can be found in Appendix A.4.

**Fig. 2:**
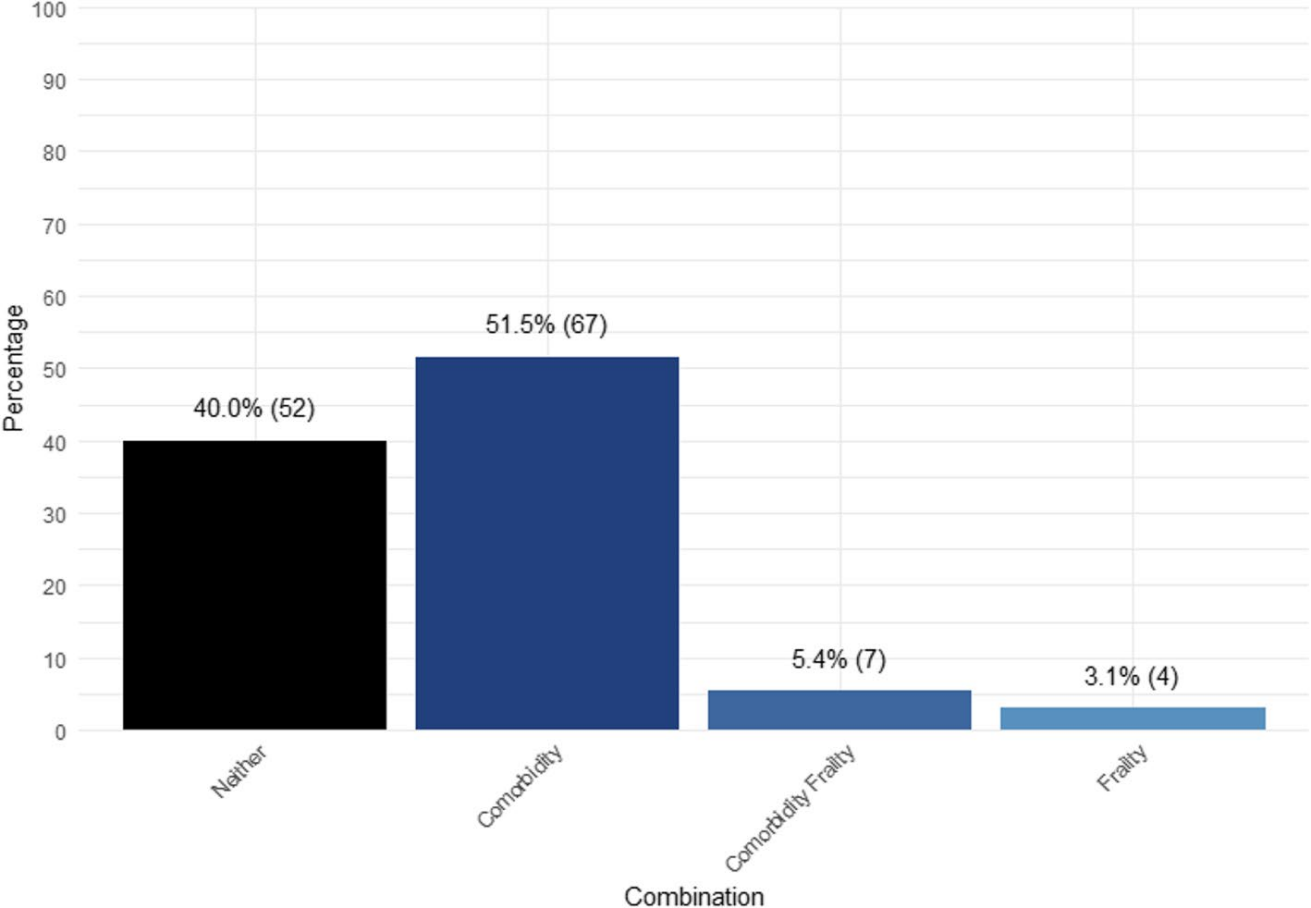
Distribution of studies by combination of assessments used (*n* = 130)

Compared to frailty assessments, the use of comorbidity assessments was more frequent, with 56.9% of all studies employing them. The distribution of studies by combination of assessments used is displayed in Fig. 2. Nearly 75% (55/74) of studies that included comorbidity assessments utilized either the Charlson Comorbidity Index or a modified version. A list of other health status assessments categorized by CGA domain is available in Appendix A.5.

**Fig. 3:**
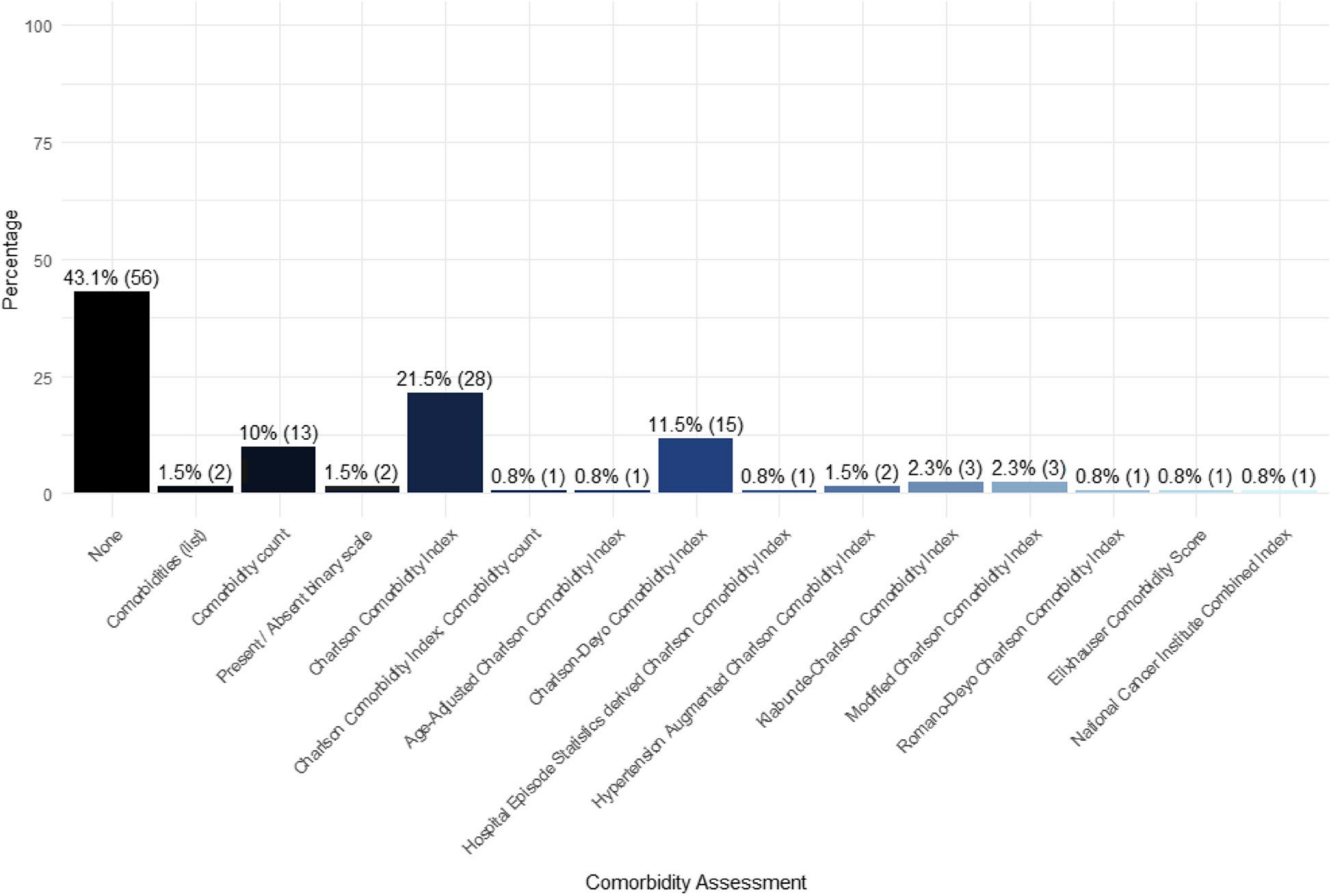
Distribution of comorbidity assessments used (*n* = 130)

Additional comorbidity assessments included the Elixhauser Comorbidity Score (*n* = 1), comorbidity counts (*n* = 13), lists (*n* = 2), and binary scales (*n* = 2). The full distribution of comorbidity assessments is shown below (Fig. 3).

**Table 2:** Overview of frailty assessments and definitions

Study ID	Frailty assessment	Definition	Domain:Test(s)	n	%Fit	%Pre- frail	%Frail	^b^AT—Tx
Battisti 2021 [9]	Geriatric assessments (score)	This novel frailty definition is based on 7 geriatric assessments, scored individually and then standardized into overall scores where patients are categorized as: Fit: 0–2; Vulnerable: 3–8; Frail: > 9	Comorbidity: CCI; Functional status: ECOG-PS, ADL, IADL; Cognition: MMSE; Nutrition: aPG-SGA; Polypharmacy: Concurrent medications excluding vitamins and minerals	2756	73.33	26.60	0.07	B—S; R
Hannoun-Levi 2021 [73]	Balducci score (frail/not frail)	According to the Balducci score patients are considered increasingly frail if they fulfill one or more of following criteria: Age > 85, Dependence in one or more ADL, presence of three or more comorbid conditions, presence of one or more geriatric syndromes. A Balducci score of 1 (fit) is assigned to an individual who has no functional dependencies, or comorbidities. A Balducci score of 2 (pre-frail) is assigned to an individual who has between one and two comorbidities and at most 1 geriatric syndrome. A Balducci score of 3 (frail) is assigned to an individual with 1 or more dependencies, more than 3 comorbidities, and more than 1 geriatric syndrome	Demographic data and social status: Age; Comorbidities: Count; Functional status: ADL, IADL; Geriatric syndromes: Dementia, Falls, Delirium, Depression, Incontinence, Osteoporosis, Neglect and abuse, Failure to thrive	157	-	-	-	B—R
Kedzierawski 2021 [86]	Geriatric 8 tool (frail/not frail)	The Geriatric 8 screening tool is composed of 8 scored questions with a total score ranging from 0–17 where scores < 14 are low risk (not frail) and scores > 14 are high risk (frail)	Demographic data and social status: Age; Functional status: Mobility, Self-health consideration; Cognition: Neuropsychological problems; Nutrition: BMI, Food intake, Weight loss; Polypharmacy: Prescription drug usage	259	-	-	-	B—S; R; H; C; T
Leo 2019 [93]	Comprehensive Geriatric Assessment (NA)	Undefined	Functional status: ADL, IADL, ECOG-PS; Cognition: Total MMSE; Depression: GDS; Nutrition: Total MNA	50	-	-	-	B—C; T
Mandelblatt 2017 [98]	Adapted Searle Deficits Accumulation Frailty Index (index)	This adapted Searle Deficits Accumulation Frailty Index is composed of 35 assessed criteria, individually rated from 0 (no deficit), 0.5–0.75 (intermediate values of deficit), or 1 (deficit present). Values for non-missing items were summed, divided by the total number of non-missing items and standardized to yield a final score between zero and one, where a higher score indicates greater frailty. Frailty scores were categorized on cut-points in the literature related to mortality outcomes as follows: Robust = 0 to < 0.2; Pre-frail = 0.2 to < 0.35; and Frail ≥ 0.35 to 1	Comorbidity: Pre-diagnosis comorbidity status for heart disease, Stroke, Diabetes, Arthritis, Rheumatism or other connective tissue disorder, Emphysema, Chronic bronchitis or asthma, Chronic liver or kidney disease, Other cancer/leukemia, Glaucoma, Cataracts or decreased vision, Blood pressure, Osteoporosis, Eyesight problems, Hearing loss; Functional status: Various ADL and IADL	1256	76.68	18.26	5.06	B – S; R; H; C
Morgan 2020 [105]	Activities of Daily Living (frail/not frail); Instrumental Activities of Daily Living (frail/not frail)	ADL dependency is measured as a proxy for frailty where being ADL independent = not frail and being ADL dependent = frail (> 1); IADL dependency is measured as a proxy for frailty where being IADL independent = not frail and being IADL dependent = frail (> 1)	Functional status: ADL, IADL	2816	-	-	-	B—S
Reeder-Hayes 2021 [120]	Faurot Frailty Index (index - quartiles)	ADL dependency is measured as a proxy for frailty, and divided into quartiles	Functional status: ADL	10,204	-	-	-	B—R; H
Ring 2021 [121]	Geriatric assessments (score)	This novel frailty definition is based on 7 geriatric assessments, scored individually and then standardized into overall scores where patients are categorized as: Fit: 0–2; Vulnerable: 3–8; Frail: ≥ 9	Comorbidity: CCI; Functional status: ADL, IADL, ECOG-PS; Cognition: MMSE; Nutrition: aPG-SGA; Polypharmacy: Concurrent medications	2811	73.25	26.68	0.07	B—C; T
Van der Plas-Krijgsman 2022 [144]	Geriatric assessments (NA)	Undefined	Demographic data and social status: Age; Comorbidity: CCI Functional status: Modified Barthel Index; Cognition: MMSE; Nutrition: BMI, MUST	-	-	-	-	B—S; R; H; C
Wyld 2021 [154]	Geriatric assessments (NA)	Undefined	Comorbidity: CCI; Functional status: ADL, IADL; ECOG-PS; Nutrition: aPG-SGA; Cognition: MMSE; Polypharmacy: Medications; ^a^Prognosis: NPI	3880	-	-	-	B—S
Yan 2021 [155]	Mian Deficits of Accumulation Frailty Index (index)	This deficits of accumulations index includes 25 items. DAFI scores ranged from 0 to 1. Patients were categorized as follows (based on prior studies): Robust: 0 to < 0.2; Pre-frail: 0.2 to < 0.35; and Frail: 0.35 to 1	Comorbidity: Chronic health conditions; Functional status: ADL, Physical function; Depression: Mental health; ^a^General health: General health, Pain interfering with work, Lots of energy	660	49.52	29.41	21.07	B—S; R

^a^: Domain not included in Comprehensive Geriatric Assessment; ^b^: AT—Tx indicates Assessment Timepoint – Treatment where B = Before Treatment, A = After Treatment, C = Chemotherapy, H = Hormone Therapy, R = Radiotherapy, S = Surgery, T = Targeted Therapy. *aPG-SGA *Abridged Patient-Generated Subjective Global Assessment;* ADL*: Activities of Daily Living; *CCI *Charlson Comorbidity Index, *ECOG*-PS Eastern Cooperative Oncology Group Performance Status, *IADL* Instrumental Activities of Daily Living, *MMSE* Mini-Mental State Examination, *BMI *Body Mass Index, *GDS *Geriatric Depression Scale, *MNA *Mini Nutritional Assessment, *NA *Not applicable, *SF*-12 Short Form-12, *MUST* Malnutrition Universal Screening Tool, *NPI *Nottingham Prognostic Index

## Discussion

This systematic review summarizes the current use of frailty assessments in observational studies investigating survival or mortality outcomes for older breast cancer patients. The findings show that less than 10 percent of these observational studies utilize frailty assessments. Additionally, there is significant variation in how frailty is defined and how patients are subsequently classified based on these definitions. It also illustrates that the majority of researchers tend to rely on less comprehensive health indicators such as comorbidity, which are often used as a substitute for frailty. The majority of frailty assessments identified in our systematic review have been previously validated [164], however, a small subset of assessments were novel, generated from combinations of individual geriatric parameters [42, 121, 144, 154], or single assessments [105]. The proportion of baseline pre-frail or frail patients captured by studies included in our review ranged from 0.07-21.07% for frail and 18.26-29.41% for pre-frail patients. While there was substantial heterogeneity in the estimates, it is clear that a high proportion of older breast cancer patients are frail. Currently there is no specific assessment recommended for use in observational studies centered on older breast cancer patients.

Frail older patients need personalized care strategies to optimize treatment outcomes and post-treatment recovery. In the clinical setting, frailty assessments are primarily useful because they enable clinicians to determine the most suitable cancer treatment for their patients while minimizing excess harm. In observational research, the primary motivations for utilizing frailty information include improving predictive and causal analyses, which can be used to inform the design of future RCTs. Interpreting the findings of observational studies becomes challenging in the absence of frailty information, as frailty has a significant impact on various health outcomes for older cancer patients. Incomplete measurements and adjustments for frailty in relevant analyses can therefore lead to confounding bias and diminish our ability to make accurate predictions or causal estimations.

A systematic review published by Wang et al [165] published in 2022, estimated that the prevalence of pre-frailty and frailty in breast cancer patients were 32% and 30%, respectively and confirmed that age is positively associated with higher levels of frailty. Another review which looked at population levels of frailty, found that frailty was higher for women compared to men [166]. Considering this information, and the possibility of ascertainment bias due to the likelihood of missing data for frail older patients, we believe the proportion of frail individuals are likely underestimated in the studies we identified. It is known that classification of patients, i.e., who is considered fit, pre-frail or frail, depends heavily on the assessment used [166], and that frailty prevalence rates exhibit less variation when arranged by definition [167]. In our review, two [42, 121] out of four [42, 98, 121, 155], studies used similar definitions for their frailty assessments and had close estimates. Estimates derived from studies which used different definitions, and different cohorts, showed much greater variability. However, the similarity could also be attributed to use of the same cohort.

Limited use of frailty assessments in observational research may stem from the overall lack of knowledge on special considerations for older adults. First, it is crucial for health care specialists in clinical practice to routinely collect this data for all older adults and to make it accessible for use in research. Second, researchers should distinguish between the health status assessments that describe vulnerabilities commonly found in older adults, as the distinctions between these may not always be clear. In particular, it’s essential to understand that frailty represents a unique dimension of aging, which sets it apart from comorbidity and disability [29, 168]. Another reason for their limited use is that much of the data in observational studies comes from healthcare databases that have been long established, and they are not required to collect data on frailty. Ideally, the assessment of frailty for older adults should be consistently and systematically conducted within clinical settings, with their integration into healthcare databases mandated as standard practice. Addressing this oversight in data repositories is essential for a comprehensive understanding of health outcomes. However, until this becomes feasible, one possible solution is to generate a frailty measure from information present in healthcare databases, which can be done with or without a reference standard [169]. For example, frailty assessments derived from electronic health records have been shown to exhibit similar performance to in-person evaluations, retain their predictive ability, and demonstrate convergent validity between research standard frailty assessments [170–172].

Many of the studies we identified, which utilized a frailty assessment, failed to classify patients and/or report levels of frailty for their study population. This was also the case for the single study [93] which assessed frailty with CGA. One difficulty with using CGA is that the information must be operationalized as an index or scale to distinguish between levels of frailty. Additionally, although CGA is meant to determine vulnerabilities comprehensively, there is debate on the best assessments to use for each CGA domain. This means there is likely variation between CGAs conducted in clinical settings. The frailty assessments we identified, including indices and scales, reflect this reality. In our review, each frailty assessment had a unique definition for frailty, and used differing sets of geriatric parameters (tests). The CGA domains captured by the parameters, however, were frequently overlapping between frailty assessments. As the classification of frailty hinges on each assessment’s definition, this makes comparing frailty across populations inherently complex. Furthermore, results on the prevalence of frailty are limited by small number of studies [42, 98, 121, 155] that used these assessments, with two studies [42, 121] using the same patient cohort.

Additionally, a group of researchers attempting to compare frailty assessments in different clinical and social settings determined that there is limited consensus among tools across both areas, implying they might assess distinct dimensions of frailty [173]. Thus, there is a compelling case for exploring frailty assessments that are specifically aligned with health outcomes which impact older breast cancer patients, aiming for a standardized approach. Adopting this perspective would acknowledge the diverse impact of frailty on different diseases, highlighting that certain tools may offer insights on specific aspects of frailty which are more relevant to this population. This would promote field specific, contextualized, and interpretable findings in future research.

Surprisingly, a majority of the studies we identified in our review use comorbidity in their analyses, but many do not consider any dimension of health status in their older population. In the absence of exhaustive data to define a frailty assessment, it is ethically and methodologically justifiable to employ alternative health assessments as surrogate indicators. However, relying on a singular, or less comprehensive health metric risks overlooking the multidimensional nature inherent to older adult health.

Four recent randomized controlled trials have assessed the effectiveness of CGA in improving post- treatment outcomes for older cancer patients [23, 25, 174, 175]. The results demonstrated that treatment decisions based on CGA reduce the incidence of toxic effects from chemotherapy and may improve rates of treatment continuation/completion and unplanned hospital admissions; however, there was no evidence for differences in overall survival or progression-free survival between patients receiving CGA based intervention and standard care. In all trials, evaluating frailty status helped physicians choose the best care strategies for their patients. Regardless, of the direct effect on survival, frail patients are more susceptible to mortality from other causes [149]. This increased susceptibility can in turn influence the extent to which patients can benefit from treatment, including the duration of survival time. In light of this information, it is important to explore the role of frailty assessments in observational studies focusing on additional metrics such as patient reported outcomes, time without symptoms, or time to treatment failure, as these may be more meaningful for older breast cancer patients [176]. Given our findings, however, it is likely that frailty assessments are also overlooked for other research outcomes as well. All things considered, we recommend frailty assessment use in clinical decision-making and along care and recovery pathways.

A strength of this review is the comprehensive search strategy used to identify target studies and a thorough evaluation of evidence through rigorous critical appraisal. To our knowledge, this is the first review to synthesize evidence to quantify and characterize the use of frailty assessments in observational studies for the older breast cancer population. Our review was limited by the narrow examination of outcomes (survival, mortality) in a short time frame. We also report recent use of frailty assessments and are therefore unable to capture time trends. Lastly, due to a lack of translation resources, we considered studies only in English, German, Spanish, and Dutch. This restriction may have potentially reduced the pool of eligible studies screened.

## Conclusion and recommendations

Frailty is an important determinant of health outcomes in older breast cancer patients. However, the majority of observational studies focusing on survival and mortality outcomes do not include frailty assessments. Missing frailty data in these studies may lead to incomplete or biased conclusions about appropriate cancer treatment. To increase their use, it is crucial to prioritize routine and standardized data collection in the clinical setting for use in health databases, and to improve education on health status assessments for researchers. To understand the use of frailty assessments more comprehensively, future research should examine the application of these assessments in studies with endpoints besides survival and mortality. By restructuring frailty measures into observational data, we can gain a better understanding of its impact and inform evidence-based guidelines to optimize patient-centered treatment in this vulnerable group of patients.

### Supplementary Information

Supplementary Material 1.

## Data Availability

The following can be acquired from the corresponding author upon reasonable request: raw and manipulated data and code used to generate material in the publication.
