# Supplementary material for: Frequency of use and characterization of frailty assessments in observational studies on older women with breast cancer: a systematic review
Source: BMC Geriatr. 2024 Jun 27;24:563. doi: 10.1186/s12877-024-05152-5 (PMC11212278; doi:10.1186/s12877-024-05152-5)
Supplement: Supplementary file 1 — Supplementary Material 1. [file 12877_2024_5152_MOESM1_ESM.pdf]

Appendix A Supplementary material

A.1 Research protocol with search strategy

A.2 List of excluded studies

A.3 Quality of included studies

A.4 Author provided frailty definitions

A.5 Alternate health status assessments

A.1 Research protocol with search strategy

Systematic Review Protocol: Frequency of use and characterization of frailty assessments in observational studies on older women with breast cancer: A systematic review

Dafne N. Sanchez[1], Marloes G.M. Derks[2], Jose A. Verstijnen[3], Dominik Menges[1], Johanneke E.A. Portielje[3], Frederiek Van den Bos[4], and Esther Bastiaannet[1]

[1]Epidemiology, Biostatistics and Prevention Institute (EBPI), University of Zurich (UZH), Zurich, Switzerland
[2]Department of Medical Oncology, Leiden University Medical Center, Leiden, The Netherlands
[3]Department of Medical Oncology, Maasstad Hospital, Rotterdam, The Netherlands
[4]Department of Gerontology and Geriatrics, Leiden University Medical Center, Leiden, The Netherlands

February 23, 2022

Background

Frailty status varies in older breast cancer (BC) patients. Several frailty assessments have been developed as a proxy measure of aging in older adults [3]. Previous studies have shown that identifying frailty is useful for risk prediction and decision-making in clinical settings [2]. Understanding the extent of use and applicability of assessments in observational studies is therefore essential to improving health outcomes in the aging population.

Aim

The aim of this review is to characterize the use, and determine the prevalence of frailty assessments in observational studies evaluating survival or mortality on older women with breast cancer (65+), concentrating on literature from the past 5 years (2017-2022).

Objectives

Primary objectives:

- Document which observational studies have been published in the last 5 years
- Quantify and characterize frailty assessments in included observational studies

Secondary objectives:

- Assess the prevalence of frailty in older breast cancer patients

Methods

Review question

How are frailty assessments used in observational studies evaluating survival or mortality for older women (65+) with breast cancer?

- Setting: The sample consists of older breast cancer patients, i.e., women (65+), participating in observational studies
- Phenomenon of interest: The phenomenon of interest is the use of frailty assessments in the context of observational studies assessing survival or mortality in older breast cancer patients
- Design: Observational studies (case-control, cross-sectional, cohort)
- Evaluation: Survival or mortality
- Research type: Peer-reviewed studies

Search strategy

We will attempt to identify all observational studies on older adults (65+) with breast cancer which report on survival or mortality published from 2017-2022, via an electronic search. The strategy will be created by the liaison librarian of the University of Zurich. Additional articles will be mined by inspecting the bibliography of relevant texts until redundancy is reached, and hand searching on Google Scholar.

The search will include articles found via the following databases:

- Cochrane Library
- Medline
- Embase

Search terms

Embase (via Elsevier):
#1 'breast cancer'/exp OR (((breast OR mamma*) NEAR/4 (cancer OR neoplasm* OR malignanc* OR tumor* OR tumour* OR carcinoma* OR angiocarcinoma* OR sarcoma* OR adenocarcinoma*)):ti,ab)
#2 'aged'/exp OR 'geriatrics'/de OR 'geriatric patient'/exp OR aged*:ti,ab OR elder*:ti,ab OR eldest:ti,ab OR old*:ti,ab OR ageing:ti,ab OR aging:ti,ab OR senior*:ti,ab OR geriatric*:ti,ab OR senium*:ti,ab
#3 'survival'/exp OR 'mortality'/exp OR 'death'/exp OR surviv*:ti,ab,kw OR death*:ti,ab,kw OR dead*:ti,ab,kw OR die:ti,ab,kw OR died:ti,ab,kw OR dying:ti,ab,kw OR mortal*:ti,ab,kw OR fatal*:ti,ab,kw OR lethal*:ti,ab,kw
#4 'cancer therapy'/exp OR 'cancer surgery'/de OR 'breast cancer'/exp/dm_dt,dm_rt,dm_su,dm_th OR 'treatment outcome'/exp OR outcome*:ti,ab,kw OR treat*:ti,ab,kw OR therap*:ti,ab,kw OR polytherap*:ti,ab,kw OR chemotherap*:ti,ab,kw OR radiotherap*:ti,ab,kw OR radiochemotherap*:ti,ab,kw OR immunotherap*:ti,ab,kw OR medication*:ti,ab,kw OR surgery:ti,ab,kw OR surgic*:ti,ab,kw OR tumorectom*:ti,ab,kw OR tumourectom*:ti,ab,kw OR (((radiation* OR irradiation* OR resect* OR remov* OR excis*) NEAR/3 (cancer OR neoplasm* OR malignanc* OR tumor* OR tumour* OR carcinoma* OR angiocarcinoma* OR sarcoma* OR adenocarcinoma*)):ti,ab,kw)
#5 ('observational study'/exp OR 'case control study'/exp OR 'cohort analysis'/exp OR 'follow up'/exp OR 'longitudinal study'/exp OR 'prospective study'/exp OR 'retrospective study'/exp OR 'cross-sectional study'/exp OR major) AND clinical AND 'study'/exp OR cohort*:ti,ab,kw OR baseline:ti,ab,kw OR multivariat*:ti,ab,kw OR (((observation* OR 'non-randomis*' OR 'non-randomiz*' OR 'nonrandomis*' OR 'nonrandomiz*' OR 'non-experiment*' OR nonexperiment* OR concurrent OR incidence OR 'follow up' OR followup OR longitudinal OR prospective OR retrospective OR 'ex-post-facto' OR 'disease frequency' OR prevalence OR consecutive OR compar*) NEAR/3 (assign* OR control* OR stud* OR research* OR design* OR group* OR matched OR analys* OR survey* OR evaluat* OR surveill* OR trial* OR questionnaire*)):ti,ab,kw) OR ((case NEAR/3 (control* OR compar*)):ti,ab,kw) OR ((case NEAR/3 (compeer* OR referent* OR referrent* OR base*)):ti,ab,kw) OR ((cross NEAR/3 sectional*):ti,ab,kw)
#6 1 AND 2 AND 3 AND 4 AND 5
#7 6 NOT [review]/lim NOT [conference abstract]/lim AND [2017-2022]/py AND ([dutch]/lim OR [english]/lim OR [german]/lim OR [spanish]/lim) NOT (('animal'/de OR 'animal experiment'/exp OR 'nonhuman'/de) NOT ('human'/exp OR 'human experiment'/de))

MEDLINE (via Ovid):
#1 exp Breast Neoplasms/ or ((breast or mamma*) adj4 (cancer or neoplasm* or malignanc* or tumor* or tumour* or carcinoma* or angiocarcinoma* or sarcoma* or adenocarcinoma*)).ab,ti
#2 exp Aged/ or Geriatrics/ or (aged* or elder* or eldest or old* or ageing or aging or senior* or geriatric* or senium*).ab,ti.
#3 exp Survival/ or exp Survival Analysis/ or exp Mortality/ or exp Death/ or (surviv* or death* or dead* or die or died or dying or mortal* or fatal* or lethal*).ti,ab,kw.
#4 ((exp Therapeutics/ or General Surgery/) and exp Breast Neoplasms/) or exp Radiotherapy/ or exp Combined Modality Therapy/ or exp Breast Neoplasms/dt, rt, su, th or exp Treatment Outcome/ or (outcome* or treat* or therap* or polytherap* or chemotherap* or radiotherap* or radiochemotherap* or immunotherap* or medication* or surgery or surgic* or tumorectom* or tumourectom*).ti,ab,kw. or ((radiation* or irradiation* or resect* or remov* or excis*) adj3 (cancer or neoplasm* or malignanc* or tumor* or tumour* or carcinoma* or angiocarcinoma* or sarcoma* or adenocarcinoma*)).ti,ab,kw.
#5 exp Observational Study/ or exp Case-Control Studies/ or exp Cohort Studies/ or exp Cross-Sectional Studies/ or clinical study/ or (cohort* or baseline or multivariat*).ti,ab,kw. or ((observation* or non-randomis* or non-randomiz* or nonrandomis* or nonrandomiz* or non-experiment* or nonexperiment* or concurrent or incidence or 'follow up' or followup or longitudinal or prospective or retrospective or 'ex-post-facto' or 'disease frequency' or prevalence or consecutive or compar*) adj3 (assign* or control* or stud* or research* or design* or group* or matched or analys* or survey* or evaluat* or surveill* or trial* or questionnaire*)).ti,ab,kw. or (case adj3 (control* or compar*)).ti,ab,kw. or (case adj3 (compeer* or referent* or referrent* or base*)).ti,ab,kw. or (cross

adj3 sectional*).ti,ab,kw.
#6 1 and 2 and 3 and 4 and 5
#7 limit 6 to (yr="2017 - 2022" and (dutch or english or german or spanish))
#8 7 not review.pt. not (animals not humans).sh.

Cochrane Library:
#1 (breast or mamma*) NEAR/4 (cancer OR neoplasm* OR malignanc* OR tumor* OR tumour* OR carcinoma* OR angiocarcinoma* OR sarcoma*)
#2 (aged* OR elder* OR eldest OR old* OR ageing OR aging OR senior* OR geriatric* OR senium*):ti,ab,kw
#3 (surviv* OR death* OR dead* OR die OR died OR dying OR mortal* OR fatal*
#4 (treat* OR therap* OR polytherap* OR chemotherap* OR radiotherap* OR radiochemotherap* OR immunotherap* OR medication* OR surgery OR surgic* OR tumorectom* OR tumourectom*):ti,ab,kw OR ((radiation* OR irradiation* OR resect* OR remov* OR excis*) NEAR/3 (cancer OR neoplasm* OR malignanc* OR tumor* OR tumour* OR carcinoma* OR angiocarcinoma*
#5 (cohort* OR baseline OR multivariat*):ti,ab,kw OR ((observation* OR non-randomis* OR non-randomiz* OR nonrandomis* OR nonrandomiz* OR non-experiment* OR nonexperiment* OR concurrent OR incidence OR "follow up" OR followup OR longitudinal OR prospective OR retrospective OR "ex-post-facto" OR "disease frequency" OR prevalence OR consecutive OR compar*) NEAR/3 (assign* OR control* OR stud* OR research* OR design* OR group* OR matched OR analys* OR survey* OR evaluat* OR surveill* OR trial* OR questionnaire*)):ti,ab,kw OR (case NEAR/3 (control* OR compar*)):ti,ab,kw
#6 1 AND 2 AND 3 AND 4 AND 5 with Cochrane Library publication date Between Jan 2017 and Dec 2022, in Cochrane Reviews

Inclusion and exclusion criteria

Articles will be included in the systematic review provided they meet the following criteria:

1. Article is (or reported on) an observational study defined here as a case-control study, cross-sectional study, or cohort study
2. Article reported solely on older females ≥ 65 years of age with all stages of breast cancer who were patients receiving active oncological treatment at the time of enrollment
3. Article is written in English, German, Dutch, or Spanish
4. Article reports on survival or mortality before or after treatment
5. Article is published within the specified 5-year period (2017-2022)

Studies were excluded based the following criteria:

1. Article is a letter, comment, conference abstract, partial text, or review
2. Article reported on a mixed population which includes individuals younger than 65 years of age, male patients, cancers besides breast cancer, and patients receiving best supportive care without active oncological treatment in the last stage of the disease
3. Article is about health technology assessment, (population) breast cancer screening, or a tool validation study
4. Article is primarily a molecular analysis (i.e. RNA, DNA, tumor structure, single cells, protein expression, biomarkers, genomic testing, gene expression etc.)

Data extraction

Titles and abstracts mined using the search strategy will be independently assessed for relevance by two review authors on Covidence [1]. The full-text versions of all suitable articles will then be uploaded to Covidence and examined by two independent reviewers for inclusion. Any disagreements on inclusion will be discussed and resolved by consensus.

Assessment of risk of bias

Risk of bias will be assessed by two separate reviewers (EB and DS) using critical appraisal tools from the Joanna Briggs Institute (JBI) appropriate for each study type. Any disagreements will be solved by consensus.

Contribution of authors

- DS led the conduct of the review, designed the review question and all forms for data extraction/collection, participated in the article screening and risk of bias assessment, and wrote the manuscript.
- EB led the conduct of the review, designed the review question, participated in the article screening and risk of bias assessments, and edited all versions of the manuscript.
- All additional authors helped to screen articles and reviewed all versions of the manuscript.

Declarations of interest

The authors have no conflicts of interest to report.

Modifications to the protocol

- The full author list and contribution of authors were added prior to the start of the review.
- The term "older adults" was changed to "older women" in the title. The title was changed from the original.
- The term "measurement" was changed to "assessment" for clarity.
- Inclusion criteria number 2 was rephrased for clarity. Originally: "Article reports solely on older females with breast cancer ≥ 65 years of age who are patients (not survivors) at the time of enrollment".
- Exclusion criteria number 2 was rephrased for clarity. Originally: "Article reports on a mixed population which includes individuals younger than 65 years of age, male patients, patients in palliative care who were being treated solely on the basis of metastatic disease, and cancers besides breast cancer".

References

[1] Covidence systematic review software, Veritas Health Innovation, Melbourne, Australia. Available at www.covidence.org.

[2] Martin A. Makary, Dorry L. Segev, Peter J. Pronovost, Karen Syin, Dora and-https://www.overleaf.com/project/62044dc8d8e90fdff6e8dd4e Bandeen-Roche, Purvi Patel, Ryan Takenaga, Lara Devgan, Christine G. Holzmueller, Jing Tian, and Linda P. Fried. Frailty as a Predictor of Surgical Outcomes in Older Patients. *Journal of the American College of Surgeons*, 210(6):901–908, June 2010.

[3] K. Rockwood and A. Mitnitski. Frailty in Relation to the Accumulation of Deficits. *The Journals of Gerontology Series A: Biological Sciences and Medical Sciences*, 62(7):722–727, July 2007.

A.2 List of excluded studies

#	Study ID	Title	Exclusion reason
1	Joris 2019	Retrospective comparison of two consecutive cohorts of adjuvant chemotherapy regimens of cyclophosphamide with either docetaxel or paclitaxel in older patients with early breast cancer	Short communication
2	Thomas 2018	An observational study investigating failure of primary endocrine therapy for operable breast cancer in the elderly	Wrong outcomes
3	Fleurier 2020	Outcome of patients with breast cancer in the oldest old (≥80 years)	Wrong patient population
4	Suen 2019	Do elderly breast cancer patients have poorer survival outcome?	Wrong patient population
5	Takuwa 2018	Overall survival of elderly patients with breast cancer is not related to breast-cancer specific survival: A single institution experience in Japan	Wrong patient population
6	Clement 2018	Role of radiotherapy in women over the age of 65 after breast conserving surgery for breast cancer: A 5-year retrospective study	Wrong patient population
7	Cao 2020	Outcomes of exclusive radiation therapy for older women with breast cancer according to age and comorbidity status: An observational retrospective study	Wrong patient population
8	Rades 2020	Individualisation of radiation therapy for older persons with secondary brain lesions from carcinoma of the breast	Wrong outcomes
9	Ma 2020	Outcome of adjuvant chemotherapy in elderly patients with early-stage, hormone receptor-positive, HER-2-negative breast cancer	Letter
10	Kim 2020	Breast cancer in elderly Korean women: Clinicopathological and biological features	Wrong outcomes
11	Jeon 2019	Optimal treatment of breast cancer in women older than 75 years: a Korea Breast Cancer Registry analysis	Wrong patient population
12	Tseng 2020	Post mastectomy radiotherapy for elderly patients with intermediate risk (T1-2N1 OR T3N0) breast cancer: A systematic review and meta-analysis	Wrong study design
13	DuMontier 2018	Health-Related Quality of Life in a Predictive Model for Mortality in Older Breast Cancer Survivors	Wrong outcomes
14	Kocik 2019	Worse survival in breast cancer in elderly may not be due to underutilization of medical procedures as observed upon changing healthcare system in Poland	Wrong patient population
15	Battisti 2021	Bridging the Age Gap in breast cancer: Impact of chemotherapy on quality of life in older women with early breast cancer	Wrong outcomes

16	Sanz 2018	Once-Weekly Hypofractionated Radiotherapy for Breast Cancer in Elderly Patients: Efficacy and Tolerance in 486 Patients	Wrong patient population
17	Yamada 2021	Systemic therapy and prognosis of older patients with stage II/III breast cancer: A large-scale analysis of the Japanese Breast Cancer Registry	Wrong patient population
18	Monib 2021	Adequate Assessment Can Affect the Management of Breast Cancer in Geriatric Population	Wrong outcomes
19	Morgan 2021	Observational cohort study to determine the degree and causes of variation in the rate of surgery or primary endocrine therapy in older women with operable breast cancer	Wrong outcomes
20	Fietz 2018	Routine treatment and outcome of breast cancer in younger versus elderly patients: results from the SENORA project of the prospective German TMK cohort study	Wrong patient population
21	Aliyu 2020	Tolerance and outcome of hypofractionated post-mastectomy radiotherapy among elderly breast cancer patients in a specialized center in Nigeria	Wrong patient population
22	Cabrera-Galeana 2020	Real-World Outcomes Among Older Mexican Women with Breast Cancer Treated with Neoadjuvant Chemotherapy	Wrong patient population
23	Lee 2017	Surgery for early breast cancer in the extremely elderly leads to improved outcomes - An Asian population study	Wrong patient population
24	Martinez-Ramos 2018	Breast cancer in octogenarian. Are we doing our best? A population-registry based study	Wrong patient population
25	Samman 2021	A population-based study of treatment patterns, 10-year recurrence and breast cancer-specific mortality in a cohort of elderly patients with breast cancer	Wrong patient population
26	Salyer 2019	Clinical characteristics and outcomes in elderly women with BRCA1 and BRCA2 mutations	Wrong condition
27	MamguemKamga 2021	Trends in endocrine therapy prescription and survival in patients with non-metastatic hormone receptor positive breast cancer treated with endocrine therapy: A population based-study	Wrong patient population
28	Schlagnitweit 2020	Impact of contemporary therapy- concepts on surgical morbidity in breast cancer patients: A retrospective single center analysis of 829 patients	Wrong patient population
29	Xu 2020	Predicting Survival Benefit of Sparing Sentinel Lymph Node Biopsy in Low-Risk Elderly Patients With Early Breast Cancer: A Population-Based Analysis	Wrong study design

30	Overgaauw 2020	Outcome and feasibility of palliative chemotherapy in very elderly patients with metastatic breast cancer	Wrong patient population
31	Holmes 2021	Cost-Effectiveness Modeling of Surgery Plus Adjuvant Endocrine Therapy Versus Primary Endocrine Therapy Alone in UK Women Aged 70 and Over With Early Breast Cancer	Wrong study design
32	Kao 2022	Short- and long-term recurrence of early-stage invasive ductal carcinoma in middle-aged and old women with different treatments	Wrong patient population
33	Vogsen 2020	Adherence to treatment guidelines and survival in older women with early-stage breast cancer in Denmark 2008-2012	Wrong patient population
34	Tzikas 2020	A comparison between young and old patients with triple-negative breast cancer: biology, survival and metastatic patterns	Wrong patient population
35	Carleton 2021	Outcomes after Sentinel Lymph Node Biopsy and Radiotherapy in Older Women with Early-Stage, Estrogen Receptor-Positive Breast Cancer	Wrong patient population
36	Ward 2017	Incidence and treatments of DCIS in octogenarians: grade matters	Wrong patient population
37	Lu 2020	Nomogram for predicting breast cancer-specific mortality of elderly women with breast cancer	Wrong study design
38	Cao 2019	The role of post-mastectomy radiotherapy in elderly patients with 1-3 positive lymph nodes breast cancer: An International Retrospective Double-Center Study	Short communication
39	Li 2017	Evaluation of the clinical benefits of adjuvant capecitabine monotherapy in elderly women with breast cancer: A retrospective study	Wrong patient population
40	Kaplan 2017	Triple-negative breast cancer in the elderly: Prognosis and treatment	Wrong patient population
41	Gray 2019	Chemotherapy effectiveness in trial-underrepresented groups with early breast cancer: A retrospective cohort study	Wrong patient population
42	Freedman 2018	Breast cancer-specific survival by age: Worse outcomes for the oldest patients	Wrong patient population
43	Allen 2022	Care of Seniors with Breast Cancer - Treatment Received and Refining Decision Making	Wrong patient population
44	Verholt 2020	Non-surgically treated older women with operable early breast cancer in Denmark	Wrong outcomes
45	Sighoko 2018	Disparity in breast cancer mortality by age and geography in 10 racially diverse US cities	Wrong patient population
46	Cabrera-Galeana 2018	Clinical characteristics and outcomes of older women with breast cancer in Mexico	Wrong patient population
47	Han 2021	Metastasis patterns and prognosis in breast cancer patients aged ≥ 80 years: A SEER database analysis	Wrong patient population

48	Patel 2021	Cancer diagnoses and survival rise as 65-year-olds become Medicare-eligible	Wrong patient population
49	Lv 2022	Metastasis patterns and prognosis of octogenarians with metastatic breast cancer: A large-cohort retrospective study	Wrong patient population
50	Mao 2017	Revisiting the impact of age and molecular subtype on overall survival after radiotherapy in breast cancer patients	Wrong patient population
51	Dillon 2021	Mortality in Older Patients with Breast Cancer Undergoing Breast Surgery: How Low is "Low Risk"?	Wrong patient population
52	Olazagasti 2021	A deep dive into CDK4/6 inhibitors: Evaluating real world toxicities and treatment paradigms in the elderly population	Wrong patient population
53	Liu 2021	Risk Stratification Model for Predicting the Overall Survival of Elderly Triple-Negative Breast Cancer Patients: A Population-Based Study	Wrong study design
54	Versteeg 2021	Predicting outcome in older patients with cancer: Comprehensive geriatric assessment and clinical judgment	Wrong patient population
55	Losada 2019	Pretreatment neutrophil/lymphocyte, platelet/lymphocyte, lymphocyte/monocyte, and neutrophil/monocyte ratios and outcome in elderly breast cancer patients	Molecular analysis
56	Schoutteten 2017	Breast cancer incidence and survival in elderly women during the 1989-2012 period: A population-based study in a French area	Wrong patient population
57	Mehta 2018	Adapting the Elixhauser comorbidity index for cancer patients	Wrong patient population
58	Sieluk 2021	Systemic therapy, survival and end-of-life costs for metastatic triple-negative breast cancer: Retrospective SEER-Medicare study of women age ≥65 years	Wrong patient population
59	Kimmick 2017	Using ePrognosis to estimate 2-year all-cause mortality in older women with breast cancer: Cancer and Leukemia Group B (CALGB) 49907 and 369901 (Alliance A151503)	Wrong study design
60	Kamaraju 2019	Are aromatase inhibitors associated with higher myocardial infarction risk in breast cancer patients? A Medicare population-based study	Wrong outcomes
61	Keim-Malpass 2020	Evaluating the Long-Term Impact of a Cooperative Group Trial on Radiation Use and Adjuvant Endocrine Therapy Adherence Among Older Women	Wrong outcomes
62	Rassu 2021	Breast surgical oncology in elderly and unfit patients: a systematic review	Wrong study design
63	Honma 2021	Clinicopathological characteristics and prognostic marker of triple-negative breast cancer in older women	Wrong patient population

64	Clifton 2019	Progression-free survival (PFS) and toxicities of palbociclib in a geriatric population	Wrong patient population
65	Bruera 2021	Systemic Lupus Erythematosus is a Risk Factor for Mortality in Elderly Patients with Early Breast Cancer	Wrong patient population
66	Bazan 2019	Assessing the Impact of CALGB 9343 on Surgical Trends in Elderly-Women With Stage I ER+ Breast Cancer: A SEER-Based Analysis	Wrong outcomes
67	Daniels 2019	Trastuzumab use in older patients with HER2-positive metastatic breast cancer: Outcomes and treatment patterns in a whole-of-population Australian cohort (2003-2015)	Wrong patient population
68	Pamoukdjian 2019	Obesity survival paradox in cancer patients: Results from the Physical Frailty in older adult cancer patients (PF-EC) study	Wrong patient population
69	Feliu 2021	Development and validation of an early mortality risk score for older patients treated with chemotherapy for cancer	Wrong patient population
70	Sprave 2020	Radiotherapy for nonagenarians: The value of biological versus chronological age	Wrong patient population
71	Hu 2021	Efficacy and Safety of Local Radiotherapy to All Oligometastatic Sites in Elderly Patients with Metachronous Oligometastatic Cancers After Initial Treatment for the Primary Tumor	Wrong patient population
72	Trabulsi 2021	Locally Advanced Breast Cancer: Treatment Patterns and Predictors of Survival in a Saudi Tertiary Center	Wrong patient population
73	Chu 2021	10-Year Survival after Breast-Conserving Surgery Compared with Mastectomy in Louisiana Women with Early-Stage Breast Cancer: A Population-Based Study	Wrong patient population
74	Sumodhee 2021	Accelerated partial breast irradiation in the elderly: 8-year oncological outcomes and prognostic factors	Wrong patient population
75	Ward 2019	Radiation Therapy Without Hormone Therapy for Women Age 70 or Above with Low-Risk Early Breast Cancer: A Microsimulation	Wrong study design
76	Takanen 2019	Full-dose intraoperative radiotherapy in older patients with early breast cancer: Analysis of eligible criteria and outcomes	Letter
77	Mariano 2017	Evaluating the association between adjuvant chemotherapy and function-related adverse events among older patients with early stage breast cancer	Wrong outcomes
78	Jiang 2019	Clinical Application of Docetaxel Combined with Lobaplatin in Adjuvant Chemotherapy of Elderly Triple-negative Breast Cancer	Language

79	Wyld 2020	Bridging the age gap in breast cancer: optimisation of survival and quality of life outcomes in older women with early breast cancer	Abstract
80	Baba 2020	A cohort study to evaluate the efficacy and safety of postoperative adjuvant therapy in HER2-positive elderly breast cancer patients (RESPECT-cohort study)	Abstract
81	Mohapatra 2017	Survival after adjuvant and neoadjuvant chemotherapy in elderly patients with triple negative breast cancer: a Cleveland Clinic experience	Abstract
82	GarciaAnaya 2019	Long-term Results of adjuvant hypofractionated radiotherapy for breast cancer in elderly patients	Abstract
83	Wenz 2016	TARGIT E(lderly) - Prospective phase II study of Intraoperative Radiotherapy (IORT) in elderly patients with small breast cancer	Wrong study design
84	Du 2005	Effectiveness of adjuvant chemotherapy for node-positive operable breast cancer in older women	Wrong inclusion date
85	Buszek 2019	Lumpectomy plus Endocrine Therapy or Irradiation in Women Age 70 Years or Older with Hormone Receptor-Positive Early Breast Cancer: an Analysis of the National Cancer Database	Abstract
86	Hughes 2004	Lumpectomy plus tamoxifen with or without irradiation in women 70 years of age or older with early breast cancer	Letter
87	Hannoun-Levi 2021	OC-0014 APBI versus very APBI in the elderly: a comparison analysis of oncological outcome and late toxicity	Abstract

A.3 Quality of included studies

Table 1: JBI quality assessment for cohort studies

Study ID	Q1	Q2	Q3	Q4	Q5	Q6	Q7	Q8	Q9	Q10	Q11	Category
Agborbesong 2020	Y	Y	Y	N	N	Y	Y	N	U	U	Y	Medium
Akushevich 2020	Y	Y	Y	Y	Y	Y	Y	N	U	U	Y	Low
Alatawi 2021	Y	Y	Y	Y	Y	Y	Y	N	U	U	Y	Low
Ali 2019	Y	Y	Y	Y	Y	Y	Y	N	U	U	Y	Low
Al-Rashdan 2021	Y	Y	Y	Y	Y	Y	Y	Y	Y	Y	U	Low
Aly 2019	Y	Y	Y	Y	N	Y	Y	Y	Y	Y	U	Low
Aytekin 2017	Y	Y	Y	N	N	Y	N	Y	Y	I	Y	Low
Battisti 2021	Y	Y	Y	Y	N	Y	Y	Y	N	N	U	Medium
Bertolo 2020	Y	Y	Y	Y	N	Y	Y	Y	U	U	Y	Low
Blanchette 2020	Y	Y	Y	Y	Y	Y	Y	N	Y	I	Y	Low
Blay Aulina 2022	Y	Y	Y	N	N	Y	Y	Y	U	U	Y	Medium
Buszek 2019	Y	Y	Y	Y	Y	Y	Y	Y	U	U	Y	Low
Cao 2018	Y	Y	Y	Y	Y	Y	Y	Y	N	U	Y	Low
Chadha 2019	Y	Y	Y	Y	N	Y	Y	Y	U	U	Y	Low
Chagpar 2017	Y	Y	Y	Y	Y	Y	Y	Y	Y	I	Y	Low
Chen 2018	Y	Y	Y	Y	Y	Y	Y	Y	Y	I	Y	Low
Chen 2021	Y	Y	Y	Y	Y	Y	Y	Y	U	U	Y	Low
Chu 2018	Y	Y	Y	Y	Y	Y	Y	Y	U	U	Y	Low
Cil 2022	I	I	Y	Y	Y	Y	Y	Y	U	U	Y	Low
Corso 2021	Y	Y	Y	Y	Y	Y	Y	Y	U	U	Y	Low
Crozier 2020	Y	Y	Y	Y	Y	Y	Y	Y	U	Y	Y	Low
Dahn 2020	Y	Y	Y	Y	Y	Y	Y	Y	Y	I	Y	Low
De Boer 2020	N	Y	Y	Y	Y	Y	Y	Y	Y	I	Y	Low
De Boer 2021	Y	Y	Y	Y	Y	Y	Y	Y	Y	I	Y	Low
De Luca 2021	I	I	Y	Y	N	Y	Y	U	Y	I	Y	Low
Derks 2018	N	Y	Y	Y	Y	Y	Y	Y	Y	I	Y	Low
De Santis 2018	Y	Y	Y	Y	Y	Y	Y	Y	Y	I	Y	Low
Downs-Canner 2019	Y	Y	Y	Y	Y	Y	Y	Y	Y	U	Y	Low
Du 2022	Y	Y	Y	Y	Y	Y	Y	N	Y	I	Y	Low
Dumontier 2017	Y	Y	Y	Y	Y	Y	Y	Y	Y	I	Y	Low
El Badri 2021	Y	Y	Y	Y	Y	Y	Y	Y	U	U	Y	Low
Enomoto 2021	Y	Y	Y	Y	N	Y	Y	Y	Y	I	Y	Low
Escott 2020	Y	Y	Y	Y	Y	Y	Y	Y	Y	I	Y	Low
Faiz 2018	Y	Y	Y	Y	Y	Y	Y	Y	U	U	Y	Low
Fattoruso 2022	I	I	Y	Y	N	N	Y	N	U	U	Y	Medium
Frebault 2022	Y	Y	Y	Y	Y	Y	Y	Y	U	U	Y	Low
Gal 2018	Y	Y	Y	Y	Y	Y	Y	Y	Y	I	Y	Low
Goldberg 2019	Y	Y	Y	Y	Y	Y	Y	Y	Y	I	Y	Low
Goyal 2019	I	I	Y	Y	Y	Y	Y	N	U	I	Y	Low
Hannoun-Levi 2021	Y	Y	Y	N	N	Y	Y	Y	U	U	Y	Medium
Haque 2017	Y	Y	Y	Y	Y	Y	Y	Y	U	I	Y	Low
Haque 2018	Y	Y	Y	Y	Y	Y	Y	Y	U	U	Y	Low
Haque 2019	Y	Y	Y	Y	Y	Y	Y	Y	U	Y	Y	Low
Herskovic 2018	Y	Y	Y	Y	Y	Y	Y	Y	Y	I	Y	Low
Hornova 2017	Y	Y	Y	Y	Y	Y	Y	N	U	U	Y	Low
Huang 2022	Y	Y	Y	Y	Y	Y	Y	Y	Y	I	Y	Low
Iglay 2017	Y	Y	Y	Y	Y	Y	Y	N	U	I	Y	Low
Janeva 2020	Y	Y	Y	Y	Y	Y	Y	Y	Y	I	Y	Low
Jhawar 2020	Y	Y	Y	Y	Y	Y	Y	N	Y	I	Y	Low
Jobsen 2019	Y	Y	Y	Y	Y	Y	Y	Y	N	U	Y	Low
Jobsen 2021	Y	Y	Y	Y	Y	Y	Y	Y	U	U	Y	Low
Kedzierawski 2021	I	I	Y	Y	Y	Y	Y	Y	U	U	Y	Low
Kinj 2018	I	I	Y	Y	N	Y	Y	Y	U	U	Y	Medium
Kinj 2019	I	I	Y	Y	N	Y	Y	Y	Y	I	Y	Low
Klint 2021	Y	Y	Y	Y	Y	Y	Y	Y	N	U	Y	Low
Kong 2018	Y	Y	Y	Y	Y	Y	Y	N	U	U	Y	Low
La Rocca 2020	Y	Y	Y	Y	Y	Y	Y	Y	Y	U	Y	Low
La Rocca 2020	Y	Y	Y	Y	Y	Y	Y	Y	Y	I	Y	Low
Leo 2019	I	I	Y	Y	N	Y	Y	Y	Y	I	Y	Low
Lin 2021	Y	Y	Y	Y	Y	Y	Y	N	N	Y	Y	Low
Liu 2021	Y	Y	Y	Y	Y	Y	Y	Y	U	Y	Y	Low
Luo 2020	Y	Y	Y	Y	Y	Y	Y	Y	U	U	Y	Low
Luo 2021	Y	Y	Y	Y	Y	Y	Y	Y	U	U	Y	Low
Mandelblatt 2017	Y	Y	Y	Y	Y	Y	Y	Y	Y	I	Y	Low
Marks 2020	Y	Y	Y	Y	Y	Y	Y	Y	U	U	Y	Low
Martin 2021	Y	Y	Y	Y	Y	Y	Y	Y	U	U	Y	Low

McKevitt 2021	Y	Y	Y	Y	Y	Y	Y	Y	N	Y	Y	Low
Mermut 2019	I	I	Y	Y	Y	Y	Y	Y	U	U	Y	Low
Merrill 2017	Y	Y	Y	Y	Y	Y	Y	U	U	U	Y	Low
Mogal 2017	Y	Y	Y	Y	Y	Y	Y	N	Y	I	Y	Low
Morgan 2020	Y	Y	Y	Y	Y	Y	Y	Y	Y	I	Y	Low
Morita 2022	Y	Y	Y	Y	Y	Y	Y	Y	Y	Y	Y	Low
Nayyar 2020	Y	Y	Y	Y	Y	Y	Y	N	U	U	Y	Low
Nichol 2017	Y	Y	Y	Y	Y	Y	Y	N	U	Y	Y	Low
Ogawa 2019	Y	Y	Y	N	N	Y	Y	Y	U	U	Y	Medium
Ojala 2019	Y	Y	Y	N	N	Y	Y	Y	Y	Y	Y	Low
Oktay 2019	Y	Y	Y	U	U	Y	Y	N	U	U	Y	Medium
Onega 2018	Y	Y	Y	Y	Y	Y	Y	Y	Y	Y	Y	Low
Park 2017	Y	Y	Y	Y	Y	Y	Y	N	U	I	Y	Low
Peng 2021	Y	Y	Y	Y	Y	Y	Y	Y	Y	I	Y	Low
Pinsky 2020	Y	Y	Y	Y	Y	Y	Y	Y	U	U	Y	Low
Poodt 2018	Y	Y	Y	Y	N	Y	Y	Y	Y	I	Y	Low
Rais 2021	N	N	Y	Y	N	Y	Y	Y	U	U	Y	Medium
Reeder-Hayes 2017	Y	Y	Y	Y	Y	Y	Y	N	U	Y	Y	Low
Reeder-Hayes 2021	Y	Y	Y	Y	Y	Y	Y	N	Y	I	Y	Low
Ring 2021	Y	Y	Y	Y	Y	Y	Y	Y	U	U	Y	Low
Schuil 2018	Y	Y	Y	Y	Y	Y	Y	Y	Y	I	Y	Low
Schwartz 2018	I	I	Y	Y	N	Y	Y	N	U	Y	Y	Medium
Showalter 2021	Y	Y	Y	Y	Y	Y	Y	N	Y	Y	Y	Low
Sieluk 2021	Y	Y	Y	Y	N	Y	Y	Y	N	Y	Y	Low
Smith-Graziani 2020	Y	Y	Y	Y	Y	Y	Y	Y	N	Y	Y	Low
Stueber 2020	Y	Y	Y	Y	Y	Y	Y	Y	Y	Y	Y	Low
Suarez-Almazor 2020	Y	Y	Y	Y	Y	Y	Y	Y	Y	Y	Y	Low
Suen 2020	Y	Y	Y	Y	Y	Y	Y	Y	U	U	Y	Low
Sumodhee 2017	N	N	Y	Y	N	Y	Y	Y	Y	I	U	Medium
Sun 2021	Y	Y	Y	Y	Y	Y	Y	Y	U	U	Y	Low
Takada 2019	Y	Y	Y	Y	Y	Y	Y	N	Y	I	Y	Low
Tamirisa 2018	Y	Y	Y	Y	Y	Y	Y	Y	N	Y	Y	Low
Tamirisa 2020	Y	Y	Y	Y	Y	Y	Y	Y	Y	I	Y	Low
Tamirisa 2021	Y	Y	Y	Y	Y	Y	Y	Y	Y	I	Y	Low
Tang 2018	Y	Y	Y	Y	Y	Y	Y	Y	U	U	Y	Low
Tang 2021	Y	Y	Y	Y	N	Y	Y	Y	U	Y	Y	Low
Tang 2022	Y	Y	Y	Y	Y	Y	Y	Y	N	Y	Y	Low
Tannenbaum 2017	Y	Y	Y	Y	Y	Y	Y	N	U	U	Y	Low
Thompson 2021	Y	Y	Y	Y	Y	Y	Y	Y	U	U	Y	Low
Tringale 2021	Y	Y	Y	Y	Y	Y	Y	N	Y	I	Y	Low
Valachis 2021	Y	Y	Y	Y	Y	Y	Y	Y	Y	I	Y	Low
Valli 2018	Y	Y	Y	N	N	Y	Y	Y	U	U	Y	Medium
Van der Plas-Krijgsman 2022	Y	Y	Y	Y	Y	Y	Y	Y	Y	I	Y	Low
Vyas 2021	Y	Y	Y	Y	Y	Y	Y	N	Y	I	Y	Low
Wang 2018	Y	Y	Y	Y	Y	Y	Y	N	U	U	Y	Low
Ward 2018	Y	Y	Y	Y	Y	Y	Y	N	U	I	Y	Low
Ward 2019	Y	Y	Y	Y	Y	Y	Y	N	U	U	Y	Low
Wasif 2019	Y	Y	Y	Y	Y	Y	Y	Y	Y	I	Y	Low
Wickberg 2018	I	I	Y	Y	N	Y	Y	Y	Y	Y	Y	Low
Wittayanukorn 2018	Y	Y	Y	Y	Y	N	Y	Y	Y	I	Y	Low
Wu 2019	Y	Y	Y	Y	Y	Y	Y	Y	U	U	Y	Low
Wu 2019	Y	Y	Y	Y	Y	Y	Y	Y	U	U	Y	Low
Wyld 2021	Y	Y	Y	Y	Y	Y	Y	Y	U	Y	Y	Low
Yan 2021	Y	Y	Y	Y	Y	Y	Y	Y	Y	I	Y	Low
Yang 2021	Y	Y	Y	Y	Y	Y	Y	Y	Y	I	Y	Low
Yuan 2020	Y	Y	Y	Y	Y	Y	Y	N	U	U	Y	Low
Zanuso 2020	Y	Y	Y	N	N	Y	Y	Y	U	Y	Y	Low
Zhao 2021	Y	Y	Y	Y	N	Y	U	Y	U	U	Y	Medium
Zhi 2019	Y	Y	Y	Y	Y	Y	Y	Y	Y	U	Y	Low
Zhong 2020	Y	Y	Y	Y	Y	Y	Y	Y	Y	I	Y	Low
Zhong 2020	Y	Y	Y	Y	Y	Y	Y	Y	U	U	Y	Low
Zhou 2018	Y	Y	Y	Y	Y	Y	Y	Y	U	U	Y	Low

Note. I: Not Applicable; N: No; U: Unclear, Y: Yes

Table 2: JBI quality assessment for case-control studies

Study ID	Q1	Q2	Q3	Q4	Q5	Q6	Q7	Q8	Q9	Q10	Category
Karanlik 2017	Y	Y	Y	Y	Y	N	N	N	Y	U	Medium

Note. I: Not Applicable; N: No; U: Unclear, Y: Yes

Table 3: JBI quality assessment for cross-sectional studies

Study ID	Q1	Q2	Q3	Q4	Q5	Q6	Q7	Q8	Category
Park 2022	Y	Y	Y	Y	Y	Y	Y	Y	Low

Note. I: Not Applicable; N: No; U: Unclear, Y: Yes

A.4 Author provided frailty definitions

Table 1: Definition of frailty as specified by Battisti et al. 2021(1) and Ring et al. 2021(2)

Definition of fitness categories. Overall scores: Fit: 0-2; Vulnerable: 3-8; Frail: ≥9.

Domain	Range	Score		
		**0**	**1**	**2**
**ECOG PS**	**0-4**	0-1	2	3-4
**ADL**	**0-10**	20	19	≤18
**IADL**	**0-8**	8	7	≤6
**Charlson comorbidity index**	**-**	0-1	-	≥2
**Prescribed medications (excluding vitamins/minerals)**	-	≤3	≥4	
**APSGA**		0-3	4-8	≥9
**MMSE**	**0-30**	≥24	20-24	<20

Note: APSGA = Abridged Patient-Generated Subjective Global Assessment; ADL = Activities of Daily Living; ECOG PS = Eastern Cooperative Oncology Group Performance Status; IADL = Instrumental Activities of Daily Living; MMSE = Mini Mental State Examination

References

1. Battisti NML, Hatton MQ, Reed MWR, et al. Observational cohort study in older women with early breast cancer: Use of radiation therapy and impact on health-related quality of life and mortality. *Radiotherapy and Oncology*. 2021;161:166–176.

2. Ring A, Battisti NML, Reed MWR, et al. Bridging The Age Gap: observational cohort study of effects of chemotherapy and trastuzumab on recurrence, survival and quality of life in older women with early breast cancer. *British Journal of Cancer*. 2021;125(2):209–219.

A.5 Alternate health status assessments

Many health status assessments serve as components of frailty, which is quantified through the Comprehensive Geriatric Assessment (CGA). Thirty-four studies made use of health status assessments, which were not specifically frailty nor comorbidity. To show the relationship between these health assessments and frailty, we classified them according to the specific domains of the CGA. A list of the 34 studies and the respective assessments is displayed below (Table 1). Eighty-eight percent of these studies assessed functional status.

Table 1: Alternate health status assessments

Study ID	Assessment(s)	CGA Domain(s)
Aly 2019	Poor performance status	Functional status
BlayAulina 2022	†Short Form (12) Health Survey	-
Cil 2022	Eastern Cooperative Oncology Group Performance Status	Functional status
DeLuca 2021	Eastern Cooperative Oncology Group Performance Status	Functional status
Downs-Canner 2019	†E-Prognosis Score	-
Dumontier 2017	†Breast Cancer-Specific Emotional Health; Cancer-Specific Psychosocial Function; Getting Out of Bed scale; Mental Health Inventory-5; Modified Social Support Scale; Physical Function Index-10	Functional status; Geriatric syndromes; Demographic and social status
ElBadri 2021	Eastern Cooperative Oncology Group Performance Status	Functional status
Fattoruso 2022	Eastern Cooperative Oncology Group Performance Status	Functional status
Hannoun-Levi 2021	Karnofsky Index	Functional status
Karanlik 2017	American Society of Anaesthesiologists Score	Functional status
Kinj 2018	Eastern Cooperative Oncology Group Performance Status	Functional status
Kinj 2019	Eastern Cooperative Oncology Group Performance Status	Functional status
Leo 2019	†Health Related Quality of Life	-
Martin 2021	Abridged Patient Generated Subjective Global Assessment; Activities of Daily Living; Eastern Cooperative Oncology Group Performance Status; Mini Mental State Examination	Functional status; Nutritional status; Cognitive status
Mogal 2017	Activities of Daily Living	Functional status
Morgan 2020	Mini Mental State Examination; Nutritional status	Cognitive status; Nutritional status
Morita 2022	Eastern Cooperative Oncology Group Performance Status	Functional status
Oktay 2019	Performance status	Functional status
Park 2017	Eastern Cooperative Oncology Group Performance Status	Functional status
Park 2022	Activities of Daily Living; †Health Related Quality of Life	Functional status
Peng 2021	Activites of Daily Living; Barthel Index	Functional status
Rais 2021	Eastern Cooperative Oncology Group Performance Status	Functional status
Ring 2021	Activities of Daily Living; Instrumental Activities of Daily Living; Abridged Patient Generated Subjective Global Assessment; Eastern Cooperative Oncology Group Performance Status; Mini Mental State Examination; Number of concurrent medications	Functional status; Nutritional status; Cognitive status; Polypharmacy
Stueber 2020	American Society of Anaesthesiologists Score; †New York Heart Association functional classification	Functional status
Suen 2020	American Society of Anaesthesiologists Score; Functional status	Functional status
Takada 2019	Eastern Cooperative Oncology Group Performance Status	Functional status
Tang 2018	Minimum Data Set - Activities of Daily Living; Minimum Data Set - Brief Interview of Mental Status; Minimum Data Set - Cognitive Performance Scale	Functional status; Cognitive status
Tannenbaum 2017	Disability Status	Functional status
Thompson 2021	American Society of Anaesthesiologists Score	Functional status

Tringale 2021	†Suemoto Index	-
Vyas 2021	Performance status	Functional status
Yan 2021	†General health status; Depressive symptoms	Geriatric syndromes
Yuan 2020	Restricted performance status	Functional status
Zhao 2021	Karnofsky Index	Functional status

Note. † Assessment does not fall into one specific CGA domain; CGA: Comprehensive Geriatric Assessment
